# Systematic review of neurophysiological assessment techniques and metrics for mental workload evaluation in real-world settings

**DOI:** 10.3389/fnrgo.2025.1584736

**Published:** 2025-04-25

**Authors:** Moussa Diarra, Jean Theurel, Benjamin Paty

**Affiliations:** National Research and Safety Institute for the Prevention of Occupational Accidents and Diseases (INRS) - Applied Sciences for Work and Organisations Department, Vandoeuvre-les-Nancy, France

**Keywords:** mental workload, cognitive load, stress, neurophysiological measures, field, autonomic nervous system, sympathetic nervous system

## Abstract

**Introduction:**

Mental Workload (MWL) is a concept that has garnered increasing interest in professional settings but remains challenging to define consensually. The literature reports a plurality of operational definitions and assessment methods, with no established unified framework. This review aims to identify objective and validated measurement methods for evaluating MWL in real-world work contexts. Particular attention is given to neurophysiological methods, recognized for their efficiency and robustness, enabling real-time assessment without disrupting operator activity.

**Method:**

To conduct this analysis, a systematic search was performed in three databases (PubMed, ScienceDirect, and IEEEXplore), covering studies published from their inception until March 30, 2023. Selection criteria included research focusing on MWL and its derivatives, as well as neurophysiological measures applied in real-world conditions. An initial screening based on titles and abstracts was followed by an in-depth review, assisted by the bibliometric software Rayyan.

**Results:**

The explored concepts, applied methods, and study results were compiled into a synthesis table. Ultimately, 35 studies were included, highlighting the diversity of measurement tools used in field settings, often combined with subjective assessments.

**Discussion:**

Furthermore, key physiological indicators such as ECG, eye data, EEG and the relationship between MWL metrics and those uses to measure stress are emphasized and discussed. A better understanding of these interrelations could refine the assessment of their respective impacts and help anticipate their consequences on workers' mental health and safety.

## 1 Introduction

The assessment of mental demands at work (i.e., mental workload, hereinafter referred to as MWL) has been the focus of extensive research in various disciplines (ergonomics, psychology, cognitive sciences, neuroscience, etc.). The common objective of these studies is to improve working conditions by considering both employees' physical capacities and cognitive resources. While the digitalization and automation of production systems have led to a reduction in purely physical demands across many industries, these transformations have also resulted in an increase in cognitive demands, particularly due to the introduction of computerized systems and automated control mechanisms.

The modernization and digitalization of companies over the past decade have consequently resulted in operators being involved in a broader range of tasks, including machine monitoring, quality control, and production strategy verification (Cohen et al., 2018). Human performance thus remains essential for maintaining quality and productivity. However, by increasing mental workload, the intensification and diversification of cognitive tasks may lead to emotional distress and negatively affect employees' psychological health (Leslie and Hutchinson, 2018; Qu, 2013). More specifically, cognitive overload can also lead to errors (Wittenberg, 2015), increase the likelihood of incidents, workplace accidents, fatigue, or musculoskeletal disorders (Das et al., 2020; Mehta, 2016; Rusnock and Borghetti, 2018), and even cause postural and coordination issues (Grobe et al., 2017; Muldner and Burleson, 2015). Similarly, an underload state can have negative effects, such as decreased performance due to a lack of attention to the task. The assessment of MWL thus emerges as a major concern for both productivity and occupational health and safety.

Certain sectors are particularly sensitive to the impact of MWL, such as air traffic control, driving, and the medical field, where errors can have critical consequences (Arico et al., 2017; Wilbanks and McMullan, 2018). In the medical sector, excessive MWL among physicians is indeed correlated with an increase in errors (Byrne, 2013; Mazur et al., 2014). In the construction industry, mental workload acts as a stressor (Umer, 2022) that can contribute to accidents due to inattentional blindness (Chen et al., 2016; Mack, 2003). In highly automated industrial environments, the difficulty and complexity of tasks, compounded by multiple human-machine interfaces, directly impact MWL levels and perceived stress among operators (Kumar and Lee, 2022).

From a theoretical standpoint, MWL remains a multidimensional and polysemic concept (Young et al., 2015). Although definitions vary across disciplines, MWL is generally described as the ratio between the cognitive resources required to perform a task and those available to the operator (Coronado et al., 2022; Heard et al., 2018; Parasuraman et al., 2008). Several studies also emphasize the influence of additional factors such as experience, age, and learning (Stanton et al., 2004). The diversity of MWL assessment methods—including subjective self-assessment, performance-based measurements, and physiological indicators—complicates the establishment of a unified research framework (Coronado et al., 2022; Heard et al., 2018).

Subjective measures, such as the NASA-TLX (Hart and Staveland, 1988), are widely used but are subject to various biases (e.g., social desirability bias, halo and horn effect, etc.). Furthermore, they provide only a retrospective assessment of the work situation (Podsakoff et al., 2003; Shakouri et al., 2018). Physiological measures, on the other hand, enable continuous and real-time monitoring of workers' mental activity by recording, for example, cardiac activity (Fallahi et al., 2016; Solhjoo et al., 2019), brain activity (Aricò et al., 2016), skin conductance (Elena and Anastasia, 2021), temperature (Murai et al., 2017), and eye movements. They provide an unbiased insight into MWL without affecting performance in real-world situations, although they can be intrusive and sometimes sensitive to environmental factors (Naismith and Cavalcanti, 2015). Finally, no single method is unanimously recognized as the most reliable for measuring MWL (Charles and Nixon, 2019). Many studies recommend combining multiple physiological measures (e.g., heart rate variability, brain activity, skin parameters, etc.) with subjective assessment methods (Lehrer et al., 2010; Sriranga et al., 2023).

Charles and Nixon (2019) also highlight the challenge of comparing laboratory and field data. For instance, heart rate variations of up to 50% have been reported in field studies, whereas in laboratory settings, they do not exceed 10% (Wilson, 1992). These findings underscore the complexity of MWL assessment and the necessity of distinguishing between laboratory and field studies. For occupational health and safety professionals and ergonomists working in real-world settings, identifying appropriate physiological measures to complement traditional assessment methods (performance metrics, questionnaires, interviews) is crucial for real-time MWL evaluation. This would enable the development of targeted recommendations to enhance workplace health and safety. Emphasis will be placed on physiological measures due to their reliability and ability to capture workers' cognitive states in real time as they respond to task demands (Charles and Nixon, 2019; Dias et al., 2019).

In light of these considerations, the objective of this study is to provide a systematic review of physiological (objective) measures used to assess MWL in real or *in situ* work environments across all fields. A side goal is to identify the different fields in which these techniques are applied to assess MWL in real-world conditions, providing occupational safety specialists and ergonomists with a comprehensive mapping of application domains. To our knowledge, this has not been done before, as existing reviews typically focus either on a specific field (aviation, driving, surgery, etc.) or include both laboratory and field studies (Kumar and Lee, 2022; Paxion et al., 2014; Wilbanks and McMullan, 2018). Which, as previously mentioned, may not be accurate for prevention specialists in real-world settings, as the transfer of these measures from the laboratory to the field is not straightforward, and correlations have been found to be low when such attempts were made (Johnston et al., 1990).

The article is structured as follows: Section 2 details the review methodology, Section 3 presents its results, and Section 4 discusses the main findings and potential future research directions.

## 2 Methods

### 2.1 Literature review and study selection

**Figure 1:**
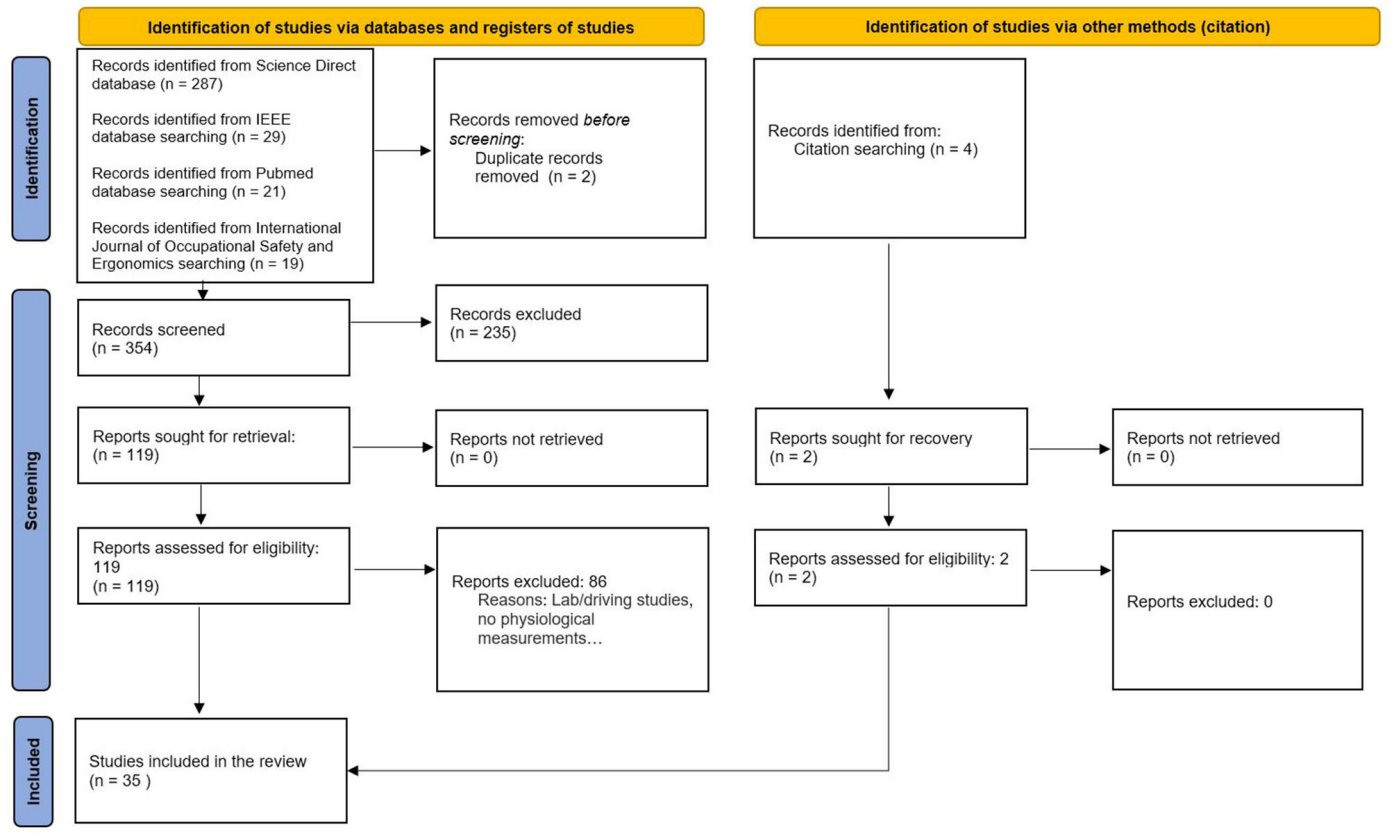
Literature selection flow diagram based on the PRISMA approach (Page et al., 2021).

We conducted a systematic review based on the PRISMA methodology (Page et al., 2021; Figure 1), designed to ensure a transparent and reproducible approach. PRISMA provides a structured guideline consisting of 27 checklist items to assist reviewers in reporting evidence with accuracy and reliability. Our objective was to identify studies that employed at least one physiological measure of MWL in real or near-real working conditions while excluding protocols conducted solely in laboratory environments with participants entirely naïve to the field.

The literature search was performed across three databases (ScienceDirect, PubMed, IEEE Xplore) from their inception until March 30, 2023. The search strings used were as follows:

- **PubMed**: “physiolog^*^ mental cognitive workload field study”.
- **ScienceDirect**: “physiology mental cognitive workload field study wearable sensor”.
- **IEEE Xplore**: (“All Metadata”:physiology) AND (“All Metadata”:mental OR “All Metadata”:cognitive workload) AND (“All Metadata”:field study).
- **For the International Journal of Occupational Safety and Ergonomics via Taylor** and **Francis Online (TandFOnline)**: “physiology mental cognitive workload field study wearable”.

We restricted our selection to studies published in English (journal articles or peer-reviewed conference proceedings). In this review, we deliberately use the terms “cognitive workload” and “mental workload” interchangeably rather than as distinct concepts. Although differences can be highlighted when compared, it is suggested that they address the same problem and thus “should be treated to mean the same thing” (Hancock et al., 2021, p. 204).

In the second stage, the titles, abstracts, keywords, and highlights were screened to assess their relevance. The inclusion criteria were:

1. Evaluation of MWL in occupational settings,
2. Use of at least one physiological measurement,
3. Investigation of a real or near-real work context,
4. Inclusion of real workers (or closely related profiles, such as cadets or specialized interns).

Studies were excluded if they were purely theoretical reviews or focused on research unrelated to actual work environments (e.g., laboratory studies with exclusively student participants or individuals distant from real workers). Additionally, studies specifically addressing driving (either in simulations or real conditions) were excluded, as they have already been extensively reviewed elsewhere (Paxion et al., 2014; Kabilmiharbi et al., 2022). For this systematic review, we employed the Rayyan tool developed by Ouzzani et al. (2016) to facilitate the screening process. Following title and abstract screening, as well as full-text analysis, 35 articles were retained for the final review, including two identified through citation search (cf. Figure 1).

### 2.2 Data collection and summary measures

We compiled the main characteristics of each article in an Excel table (publication type, study context, physiological measures used, potential use of subjective measures, etc.). This allowed for a descriptive analysis (chronological distribution of publications, preferred application domains, most commonly used techniques).

## 3 Results of the literature review

Various physiological techniques are employed in the field for assessing MWL, and the objective of this review is to identify these techniques as comprehensively as possible. Studies indicate that MWL leads to modifications in the autonomic nervous system (ANS), which notably regulates stress responses (Causse et al., 2010; Fairclough et al., 2005; Kurniawan et al., 2013). Among the most frequently used markers are heart rate (HR) and its variability (HRV), respiratory rate (RR), skin conductance (GSR, EDA), oculomotor data (pupil diameter), electroencephalography (EEG), and functional near-infrared spectroscopy (fNIRS; Sriranga et al., 2023). Table 1 summarizes the techniques and metrics retained in this review for MWL assessment in real-world conditions. This section first presents a descriptive analysis, followed by a detailed examination of each method used, highlighting their most relevant metrics, advantages, and limitations. Indeed, we first compiled the main characteristics of each article in an Excel table (publication type, study context, physiological measures used, potential use of subjective measures, etc.). This allowed for a descriptive analysis, including the chronological distribution of publications, preferred application domains, and the most commonly used techniques.

**Table 1:** Techniques and dependent variables used to assess MWL in the context of field studies.

**Techniques**	**Concept studied**	**DVs**	**Results**	**References**	**Domain**
**ECG/Salivary measures/EMG/EYE/ST/respiration/EDA/video/questionnaires**	Mental workload (MWL)	HR (average work pulse)	Increased heart rate with increased workload	(Jung and Jung, 2001)	Industry
	MWL	HR, HRV features, EMG amplitude	Increased HR, LF/HF, EMG	(Fallahi et al., 2016)	Traffic density monitoring
			Decreased SDNN, RMSSD, pNN50		
	MWL	HRV, salivary cortisol	pNN50, RMSSD decreased, LF/HF increased. HR, Mean RR, SDNN, HRV Index, TINN, Cortisol (N.S)	(Cinaz et al., 2013)	Office work
	MWL/stress	HRV, salivary NO3 concentration	Increased LF/HF and salivary NO_3_ concentration. HR (N.S)	(Kitamura et al., 2016) (Murai et al., 2017)	Maritime
	MWL/stress	HR, HRV, Breathing rate, ST, PD, EDA	Can be validly used to evaluate cognitive states (MWL) & improve the interaction between operators and industrial workplaces. HR, PD increase; HRV, fixation/sacadic frequency decrease	(Peruzzini et al., 2017, 2020) (Brunzini et al., 2021a)	Industry
	CL (cognitive load)/stress	HRV, EDA (mean SCL, amplitude SCR peaks), motion pattern (head pose, skeleton tracking)	LF/HF increased with high MWL. Both tonic and phasic components revealed a significant main effect of the load condition	(Lagomarsino et al., 2022)	Industry
	CL	HRV, IBI, Shannon entropy	Decreased in Shannon entropy & IBI (interbeat intervals) associated to high cognitive workload	(Dias et al., 2019)	Surgery
**Eye data/pupillometry/EEG/questionnaire**	MWL/error	PD	Increased PD	(Srinivasan et al., 2019)	Chemical plant
	CWL (cognitive workload)	PD, blink frequency, gaze metrics (fixation, entropy, saccades)	Greater workload involved less blinks, reduce fixation rate and increase pupil size/fixation duration/gaze entropy.	(Naik et al., 2022)	Surgery
	CWL	Fixation duration, fixation counts, pupil size	Increased fixation (duration, count) during high workload situation (during travel). Significant PD changes during task (decrease between pallet loading/unloa-ding activities and travel loaded)	(Ulutas and Firat Ozkan, 2019)	Warehouse forklift driver
	MWL	Fixation frequency, fixation duration, saccade duration, saccade amplitude, fixation/saccade ratio	Fixation duration/fixation frequency/saccade amplitude/fixation-saccade ratio increased with workload and saccade duration decreased with high workload.	(Das et al., 2020)	Construction
	MWL/engagement	Mean PD, gaze entropy, EEG (engagement index)	Gaze entropy and engagement index both showed a significant negative correlation with performance during surgeon task. Pupil diameter (PD) was not significant (N.S.).	(Wu et al., 2021)	Surgery
	MWL	Eye gaze (fixation, saccade, entropy), PD	Bigger pupil diameter indicates higher mental workload	(Zheng et al., 2022)	Manufacture, logistic
**EEG/EDA/PPG/questionnaires**	MWL	EEG spectral features: Frontal & Parietal Theta, parietal alpha frequency bands (Workload index)	Increased theta band (frontal), decreased alpha over parietal brain areas: increased EEG workload index EEG workload index highly correlate to subjective	(Aricò et al., 2015) (Aricò et al., 2016)	Air traffic control
	CL	Features extract from physiological signals (EEG: alpha, beta bands; PPG, EDA) classified with NASA TLX questionnaire + predictive model (deep neural network)	EEG predict cognitive load with 72% accuracy, PPG and EDA led to 60% accuracy. EEG+PPG+EDA led to 86% accuracy	(Shayesteh et al., 2023) (Liu et al., 2021)	Construction
	MWL	EEG time frequency analysis, engagement index (alpha, beta, theta)	Engagement index reflect mental workload, it increases when workload is high. Frequency bands can be used to assess workload in field study	(Chen et al., 2016) (Saedi et al., 2022)	Construction
	MWL	EEG frequency bands: Delta, theta, alpha, beta, gamma; Signal power measurements, phase locking value	Higher brain activity in the beta2/theta bands during more demanding task. Significant brain activation in gamma rhythms reported.	(Kosti et al., 2018)	Office Work
	MWL	Power spectral density of theta (θ) waves	Positively correlated to workload	(Iqbal et al., 2020)	Chemical process control room
**EDA/ECG/EEG/ST/respiration/eye/questionnaire**	MWL	Average skin conductance	Increased skin conductance activity during high workload phase	(Elena and Anastasia, 2021)	Aviation
	MWL	Skin conductivity response, mean respiration rate, fixation duration, pursuit distance, saccadic amplitude	Machine learning framework (physiological/subjective data) prediction of operator performance reach 75%−83% accuracy	(Zhang et al., 2020)	Nuclear power plant control room
**Salivary or biochemical indices/ST/ECG**	MWL	HRV, Salivary amylase, facial (nasal) temperature	RRI, salivary biomarkers, and nasal temperature suggested as relevant indicators; however, no specific directional relation to MWL is reported. Increased salivary NO3 concentration	(Murai, 2017) (Kitamura et al., 2016)	Maritime
	MWL	Salivary cortisol	Salivary cortisol not appropriate to evaluate MWL (N.S)	(Zoaktafi et al., 2020)	Power Plant
	MWL	Salivary cortisol, HRV	Cortisol (N.S), pNN50, RMSSD decreased, LF/HF increased. HR, Mean RR, SDNN, HRV Index, TINN.	(Cinaz et al., 2013)	Office work
**PPG**	CL	IBI, HR, pNN50, pNN20	HR increase, IBI, pNN50, pNN20 decrease with high workload (Wang et al., 2022)	(Wang et al., 2022)	Aviation
**fNIRS/questionnaires**	MWL	HbO_2_ (oxy-hemoglobin), HbR (deoxy-hemoglobin)	Increased prefrontal oxygenation	(Ayaz et al., 2012)	Air traffic control
	MWL	Oxygenated (O_2_Hb), deoxygenated (HHB) hemoglobin	Increased O_2_Hb, decreased HHb for high cognitive task	(Midha et al., 2021)	Office work
	MWL	HBO (oxygenated hemoglobin), HBR (deoxygenated hemoglobin), Hb (total hemoglobin)	Only HbO analyzed; increased HbO in right-lateral PFC with higher task load	(Fan and Yang, 2023)	Maritime
**Speech analysis**	MWL/Stress/Fatigue	Voice intensity (RMS voice energy, loudness mean, median), F0, Mel Frequency Cepstral Coefficients, jitter, number of voiced segments per second and mean voiced/unvoiced segment lengths in seconds.	Significant increase in multiple speech features during high MWL, particularly loudness (mean, median), F0, spectral features (MFCCs, jitter), and speech rate metrics (number and mean of voiced/unvoiced segments).	(Cosić et al., 2019)	Air traffic control

### 3.1 Descriptive analysis

This part provides a descriptive analysis of the 35 articles identified during the literature search, considering: the evolution of publications over time per field of application and year, the distribution of techniques used and the representativeness of each sector.

### 3.2 Temporal evolution of publications and fields of application

**Figure 2:**
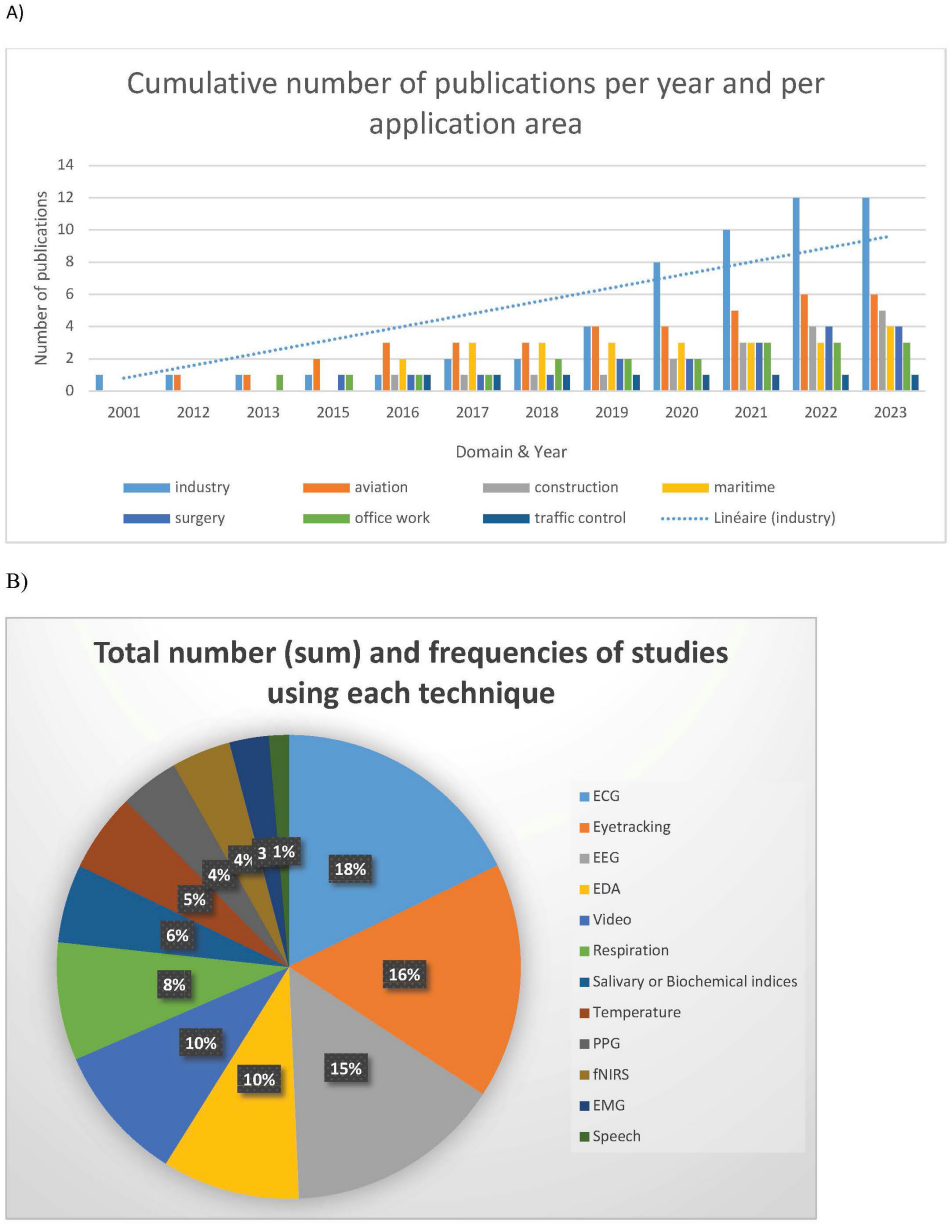
Descriptive analysis of the selected publications. **(A)** Cumulative number of publications per year and by sector of application. **(B)** Distribution of techniques used in studies.

Figure 2A presents the cumulative number of publications per year and by sector of application. Most research (~70%) that evaluated MWL using physiological measurements in field conditions was published after 2016, reflecting the recent rise and democratization of portable technologies (glasses, non-invasive sensors, etc.).

The analysis reveals that the most represented sectors are industry (34%), aviation (17%), construction (14%), maritime (12%), medical (11%), office work (9%), and traffic control (3%). Temporal evolution is observed across all sectors.

### 3.3 Measurement methods

The most frequently employed techniques for assessing MWL include ECG (and heart rate variability analysis), eye-tracking, EEG, EDA (electrodermal activity), respiration, skin temperature, EMG, fNIRS, and voice analysis. ECG, eye-tracking, and brain signals (EEG, fNIRS) constitute nearly half of the identified methods (Figure 2B). When examining the frequency of occurrence of techniques within each domain, it appears that specific techniques are prioritized in different fields, as illustrated in Table 2. This table provides insights into the techniques applicable in field settings for evaluating MWL across various occupational sectors. However, these techniques are not exclusive to any single domain.

**Table 2:** Technique used by order of importance (number of occurrences) in each application area.

**Domain**	**1**	**2**	**3**	**4**	**5**	**6**	**7**	**8**
Industry	Eye data (7)	ECG (5)	Video (5)	EDA (4)	Respiration (4)	Temperature (2)	EEG (2)	Salivary cortisol (1)
Aviation	EEG (3)	ECG (2)	EDA (2)	Speech analysis (1)	EMG (1)	fNIRS (1)	PPG (1)	Temperature (1)
Construction	EEG (4)	EDA (1)	Eye data (1)	PPG (1)				
Maritime	ECG (3)	Salivary amylase (1)	Salivary NO_3_ (1)	Temperature (1)				
Surgery	EEG (2)	Eye data (2)	Video/audio (1)	ECG (1)				
Office work	Salivary cortisol (1)	ECG (1)	EEG (1)					
Trafic control	ECG (1)	EMG (1)						

### 3.4 Electrocardiogram (ECG) and heart rate variability (HRV)

The electrocardiogram (ECG) measures the electrical activity of the heart. It is the most widely used method for assessing mental workload (MWL), both in laboratory settings (Charles and Nixon, 2019) and in real-world conditions. The majority of studies included in this review employ ECG. ECG analysis encompasses heart rate (HR) and heart rate variability (HRV; Hermans et al., 2014).

Heart rate (HR), defined as the number of beats per minute, is considered by Jung and Jung (2001) to be the simplest and most reliable indicator of MWL. These authors tested the validity of an overall workload (OWL) measure in 28 workers from nine different departments in the machine processing industry, recording heart rate using a pulse sensor placed on the earlobe. They calculated the average (WP) and relative work pulse (% RWP) by determining the difference between baseline heart rate (resting HR) and heart rate during the task. Regression analysis and classification enabled them to distinguish different HR levels based on workload. For example, an HR of 60–70 bpm (0–10 WP) is considered a resting value or baseline (Grandjean, 1980), while 70–100 bpm (10–40 WP) corresponds to low workload, 100–125 bpm (40–65 WP) to moderate workload, 125–150 bpm (65–90 WP) to high workload, 150–175 bpm (90–115 WP) to very high workload, and >175 bpm (>115 WP) to extremely high workload. Notably, this study assesses overall workload (both physical and mental), in line with the observation that HR increases in response to both physical and cognitive demands (Grandjean, 1980; Green et al., 1986). It is worth noting that this study includes a strong physical component; nevertheless, it has the merit of proposing threshold values for the evaluation of both mental and physical workload. This can be particularly valuable for field practitioners, as both components, mental and physical, are often simultaneously at play in many work environments.

Furthermore, HR elevation is associated with increased stress levels (Kaklauskas et al., 2011), reflecting the close relationship between MWL and stress. Several studies indicate that these two mental states are highly interconnected and mutually influence each other (Sanders, 1983; Yerkes and Dodson, 1908). It is therefore recommended to assess stress levels concurrently when investigating MWL (Alsuraykh et al., 2019).

Heart rate variability (HRV) is currently one of the most studied indicators for assessing mental stress and appears more sensitive than HR alone. HRV refers to variations in successive R-R intervals on the ECG and can be analyzed in the time, frequency, and non-linear domains. It can be measured over long periods (24 h), short periods (5 min), or very short periods (<5 min). When faced with a stressful event, the sympathetic nervous system is activated, while the parasympathetic system withdraws, leading to characteristic changes in HRV components. Temporal metrics include mean R-R interval duration (RRI), the standard deviation of R-R intervals (SDRR), the square root of the mean squared differences of R-R intervals (RMSSD), and the proportion of NN50 to the total number of NN intervals (pNN50). Frequency-domain metrics often include high-frequency (HF) power and the low-frequency to high-frequency (LF/HF) power ratio. In non-linear analysis, entropy indices are commonly used. These metrics exhibit significant variations in both high workload situations and stress-inducing contexts (Castaldo et al., 2015).

With regard to MWL specifically, Fallahi et al. (2016) evaluated 16 urban traffic control center operators, using subjective (NASA-TLX) and physiological (ECG, EMG) measurements. They observed that increased traffic density (resulting in greater monitoring workload) was associated with increased heart rate (HR), modifications in HRV components (increased LF/HF ratio and decreased SDNN, RMSSD, pNN50), and an increase in EMG amplitude. The authors concluded that higher MWL contributes to mental fatigue and stress, negatively impacting operators' mental health.

Studies highlight:

- An increase in mean RRI in maritime environments (Murai, 2017),
- An increase in the LF/HF ratio during high-workload tasks, including traffic control (Fallahi et al., 2016), office work (Cinaz et al., 2013), maritime navigation and port coordination (Kitamura et al., 2016; Murai et al., 2017), and industrial activities (Lagomarsino et al., 2022),
- A decrease in parasympathetic HRV indicators (RMSSD, pNN50, SDNN) in office tasks (Cinaz et al., 2013) and traffic control (Fallahi et al., 2016),
- A reduction in non-linear HRV parameters (e.g., Shannon entropy) during real-life surgical procedures (Dias et al., 2019).

The review indicates that the ECG-derived measures of interest, which have demonstrated effectiveness in assessing MWL in real-world conditions, are: HR, mean RRI, RMSSD, pNN50, SDNN, LF/HF, and entropy measures. Thus, these measures can be used for an effective assessment of MWL in real-world settings.

### 3.5 Eye-tracking and oculometric measures

Ocular data (pupil diameter, blink frequency, fixations, etc.) are also reliable indicators of increased mental workload (MWL). In recent years, oculomotor measurements (e.g., via smart glasses) have become more prevalent due to their ease of use and accessibility (Tao et al., 2019). Among the most commonly used indicators are the number and duration of blinks, eye gaze (fixation duration), and pupil diameter (PD).

Pupillometry, the measurement of pupil diameter, is considered a reliable method for assessing MWL in both laboratory and real-world conditions (Fan et al., 2020; Tao et al., 2019). In industrial environments, several studies have shown that increased pupil dilation is correlated with higher MWL (Brunzini et al., 2021a,b; Peruzzini et al., 2017, 2020). This phenomenon is influenced by a noradrenergic system known as the Locus Coeruleus (LC), which acts as an inhibitory mechanism of the parasympathetic oculomotor system (Laeng et al., 2012). The LC operates in two modes: tonic (environmental exploration and novelty detection) and phasic (focused processing of relevant stimuli—more complex tasks result in greater pupil dilation; Aston-Jones and Cohen, 2005). Pupil dilation is thus linked to mental effort, task difficulty, and physiological arousal levels (Rodríguez et al., 2015). It also reflects cardiac variations (Murata and Iwase, 2000) and is positively correlated with error rates, indicating higher MWL, as demonstrated by Gao et al. (2013) in a nuclear power plant task simulation.

Blinks and eye gaze metrics are also utilized for MWL estimation. In their review of oculomotor measures in surgery, Naik et al. (2022) noted that increased cognitive load is associated not only with pupil dilation but also with a decrease in blink frequency, consistent with previous findings in aviation (Bednarik et al., 2018; Veltman and Gaillard, 1996; Zheng et al., 2012). They also observed that experts (unlike novices) exhibit fewer fixations but with longer durations. Furthermore, gaze entropy, i.e., the uncertainty in gaze position at a given moment, tends to increase as task complexity and cognitive demand rise, although some studies suggest divergent results (Allsop and Gray, 2014; Di Nocera et al., 2007; Di Stasi et al., 2016).

In summary, high mental workload tasks are generally associated with:

- Increased pupil diameter (Naik et al., 2022; Srinivasan et al., 2019; Zheng et al., 2022),
- Longer fixation duration and higher fixation frequency (Das et al., 2020; Ulutas and Firat Ozkan, 2019),
- Greater saccade amplitude and a higher fixation-to-saccade ratio,
- Shorter saccade duration (Das et al., 2020),
- Fewer blinks,
- Increased gaze entropy (Naik et al., 2022; Wu et al., 2021).

Most studies show statistically significant distinctions between different MWL levels based on pupil diameter, fixation frequency and duration, and blink rate and duration (Das et al., 2020). These measures can be used alongside entropy measures to evaluate MWL.

### 3.6 Electroencephalography (EEG)

Electroencephalography (EEG) is a direct tool for exploring brain activity. EEG records electrical brain activity. Frequently studied parameters include mean amplitude, mean amplitudes of Event Related Potential (ERP) components, mean spectral power in each frequency band, and power ratios (Alberdi et al., 2016).

Aricò et al. (2015) calculated a MWL index based on frequency bands associated with MWL (frontal and occipital theta, parietal alpha), integrating it into a classification model (Aricò et al., 2014; Borghini et al., 2016). Machine learning techniques applied to EEG are widely used for MWL evaluation (Aricò et al., 2014; Kohlmorgen et al., 2007), although the need for frequent recalibration may limit their operational use (Aricò et al., 2015). Despite this, Aricò et al. (2016) demonstrated that real-time MWL monitoring is feasible for air traffic controllers in highly realistic simulation tasks by analyzing frontal theta and parietal alpha bands. Increased MWL is associated with higher frontal theta power and lower parietal alpha power, resulting in an EEG workload index that correlates strongly with subjective assessments.

In the construction industry, Shayesteh et al. (2023) used a machine learning approach combined with physiological measures (EEG, EDA, PPG) to evaluate MWL during human-machine collaboration tasks. Their EEG-based model achieved 72% accuracy, PPG and EDA 60%, and the EEG+PPG+EDA combination 86%. Liu et al. (2021) also confirmed the effectiveness of EEG (temporal and frequency domains: alpha, beta, gamma) in predicting MWL with high accuracy (81.91%) in human-robot collaboration contexts.

Other studies validate the relevance of *in situ* EEG for MWL assessment, such as in construction (Saedi et al., 2022). Chen et al. (2016) demonstrated that an EEG engagement index [beta power/(alpha power + theta power)] increases under high MWL conditions. In programmers, Kosti et al. (2018) observed increased theta and beta activity, linked to higher cognitive effort, working memory, and concentration (Jensen and Tesche, 2002). Additionally, Iqbal et al. (2020) reported that theta wave intensity increases with workload, and alpha activity correlates with arousal and workload: “a decrease in power spectral density of α is associated with an increase in arousal, mental load, stress, and anxiety” (Iqbal et al., 2020, p. 5).

EEG has the advantage of providing a direct measure of neural processes compared to indirect techniques measuring blood flow or metabolic activity (fMRI, fNIRS). It is highly sensitive to changes in cognitive states and task difficulty (Antonenko et al., 2010).

The results from studies using EEG indicate that the metrics that have proven effective for assessing MWL in real-world settings include the EEG workload index, which increases as MWL increases (Aricò et al., 2015; Chen et al., 2016), as well as neuronal activity measurements in the frontal region, specifically theta and beta bands, and alpha activity over the parietal brain, which also appear to be reliable measures of MWL in the field (Kosti et al., 2018; Aricò et al., 2016).

### 3.7 Electrodermal activity (EDA)

Sweating after a stressful event is one of the manifestations of sympathetic nervous system (SNS) activation in a situation of high-arousal. Electrodermal activity (EDA), also referred to as Galvanic Skin Response (GSR) or Skin Conductance Response/Activity (SCR/SCA), is used to measure this physiological response. EDA is defined as a change in the electrical properties of the skin. Since it is solely regulated by the SNS (without parasympathetic innervation of the sweat glands), EDA is considered a “pure” index of physiological arousal. In addition to GSR levels, EDA comprises tonic and phasic components (SCR/SCL) and the mean, maximum, or minimum amplitude of skin conductance peaks (Lim et al., 1997). It has been widely used for a long time to quantify various cognitive states such as stress and mental workload (Setz et al., 2010).

In our corpus, seven studies employed EDA to assess MWL. Elena and Anastasia (2021) examined flight simulator operators under normal and degraded conditions, highlighting an increase in skin conductance (average SC amplitude) during high-MWL flight phases. Similarly, Lagomarsino et al. (2022) developed a framework to analyze cognitive load in industrial assembly tasks using video, ECG (HRV for mental effort), and EDA (for stress). In this study, MWL and stress were measured simultaneously through physiological sensors: the tonic (SCL) and phasic (SCR) components of conductance showed significant variations based on workload intensity (mean SCL value and mean amplitude of SCR peaks). Shayesteh et al. (2023) assessed the cognitive load of masons during human-robot interaction in a virtual training environment. EEG, EDA, and PPG were integrated into a deep neural network (DNN) to estimate MWL: the EEG+EDA+PPG combination achieved 86% accuracy, compared to 60% with PPG and EDA alone, and 72% with EEG alone.

Brunzini et al. (2021b) proposed a protocol combining multiple physiological data sources (EDA, ECG, pupillometry, video, respiration) with subjective evaluations (NASA-TLX, NAS stress scale) to differentiate stress and MWL. Their assessment validated a workload evaluation model for industrial operators and underscored its potential application by designers and engineers in workload assessment and occupational disease prevention.

Zhang et al. (2020) also introduced a machine learning framework to predict employee performance, including MWL, based on physiological (electrodermal response, mean respiratory rate, eye fixation duration, eye saccade amplitude) and subjective (Halden Task Complexity Scale—HTCS) measures. Their model achieved an accuracy between 75% and 83% using these data (physiological MWL assessment techniques: eye-tracking, SCR), integrated within an SVM model.

These studies demonstrate that electrodermal activity is a viable measure for assessing mental workload. Therefore, key metrics of interest for field evaluation include average SC amplitude, mean SCL value and mean amplitude of SCR peaks (Elena and Anastasia, 2021; Lagomarsino et al., 2022).

### 3.8 Respiration

Respiration has also been considered a relevant parameter in response to increasing task difficulty. In our sample, six articles used respiration as an MWL indicator in real-world conditions, often in conjunction with other physiological measures. The literature emphasizes that the most pertinent respiratory index is respiratory rate, commonly measured via a chest strap or inferred from other physiological signals (Kuo and Chen, 2022). Several studies show an increase in respiratory rate as task complexity increases, particularly in air traffic control settings (Backs et al., 2000; Brookings et al., 1996). This increase is attributed to heightened metabolic demands associated with the effort required by the task (Roscoe, 1992).

The study by Brunzini et al. (2021b), previously discussed, incorporated respiratory monitoring and demonstrated that increased mental effort led to an increase in respiratory rate and a decrease in breathing depth (Roscoe, 1992). Additionally, Peruzzini et al. (2017, 2020) integrated respiratory measurements along with other indicators (ECG, skin temperature, posture, oculomotor parameters) to assess MWL, stress, and fatigue. It appears that respiratory rate and breathing depth are metrics of interest for monitoring mental workload in the workplace.

### 3.9 Hormonal indicators (cortisol, alpha-amylase)

Cortisol is a hormone secreted by the hypothalamic-pituitary-adrenal (HPA) axis and plays a role in the body's response to stress. In the face of acute stressors, HPA activity rapidly increases, leading to a sharp rise in cortisol levels (Chrousos, 2009). Cinaz et al. (2013) measured MWL during routine office tasks using subjective (NASA-TLX), objective (heart rate variability), and salivary cortisol indicators, as well as performance-based measures. Although some participants with high MWL showed elevated salivary cortisol, no significant overall differences were observed across different workload periods. The authors suggest that variations in salivary cortisol are more pronounced in response to uncontrollable and social-evaluative stressors (Dickerson and Kemeny, 2004). Zoaktafi et al. (2020) also examined the relationship between MWL and salivary cortisol in power plant technicians, finding that despite high MWL levels, no correlation was observed between salivary cortisol and subjective evaluations (NASA-TLX). According to these studies, salivary cortisol may not be an appropriate physiological assessment method for MWL, as it is highly dependent on individual circadian rhythms, as well as participants' levels of fatigue, burnout, exhaustion (Chida and Steptoe, 2009; Ying et al., 2011).

Recently, salivary alpha-amylase (sAA) has emerged as a novel biomarker for psychosocial and acute stress responsiveness within the sympathetic-adrenomedullary (SAM) system (Nater and Rohleder, 2009). It has also been used for MWL assessment in field conditions. Murai (2017) combined salivary amylase/nitric acid measurements with physiological parameters (heart rate variability, nasal facial temperature) to assess MWL in a maritime bridge crew. Kitamura et al. (2016) evaluated salivary NO_3_ concentrations, as nitric oxide plays a role in various physiological processes (Caramia et al., 1962). The advantage of this biomarker is that it reflects endocrine rather than autonomic nervous system activity. The findings of Kitamura et al. (2016) aligned with results obtained from R-R interval (RRI) analysis, further validating this approach. However, in field conditions, saliva sampling may be more complex and prone to failure compared to simpler heart rate measurements.

Regarding hormonal indicators, salivary alpha-amylase and salivary NO3 emerge as metrics of interest that can be used to assess mental workload in the workplace.

### 3.10 Skin temperature

Facial skin temperature (ST) is also described as an indicator of MWL. Several studies have observed a correlation between mental load and a decrease in nasal temperature (Marinescu et al., 2018; Murai et al., 2008; Or and Duffy, 2007), measured via infrared thermography. According to Or and Duffy (2007), this decrease is explained by vasoconstriction linked to stress or a negative emotion, under the influence of the sympathetic nervous system (Wallin, 1981). Shah et al. (2020) also observed, during a Stroop test, a more pronounced vasoconstriction than during a memory task (N-back) mobilizing MWL (Khaksari et al., 2019). This study thus demonstrates that, like stress, high MWL can trigger vasoconstriction and, consequently, a drop in body temperature.

Similar to other indicators previously discussed, variations in skin temperature are associated with states of stress and anxiety (McFarland, 1985). Alberdi et al. (2016) also point out that these variations result from localized changes in blood flow, which depend on the activity of the autonomic nervous system (ANS). However, findings sometimes diverge between individuals. The analyzed studies generally focus on mean, minimum, maximum, or standard deviation values of skin temperature. The slope of temperature variation is also used in order to reveal transient temperature changes (Barreto et al., 2007).

In the maritime field, Murai (2017) measured the facial (nasal) temperature of various crew members (captain, duty officer, helmsman, pilot) and found that, when combined with HRV and biochemical indices (amylase/nitric acid), it serves as a reliable indicator of MWL. Murai et al. (2008) had already noted a decrease in nasal temperature and an increase in the LF/HF ratio as workload increased. Skin temperature is also used in field settings to quantify MWL in industry (Peruzzini et al., 2017, 2020) and aviation (Elena and Anastasia, 2021).

Peripheral vasoconstriction associated with increased MWL can therefore induce variations in skin temperature. Variation in nasal temperature may serve as a reliable indicator of changes in mental workload, particularly when combined with other measures.

### 3.11 Photoplethysmography (blood volume pulse)

PPG is an optical technique that enables the acquisition of a signal related to peripheral blood volume pulse (BVP). The PPG waveform has been shown to have a good correlation with the blood pressure waveform (Xing et al., 2019). It can also be used to estimate heart rate variability (HRV). Commonly analyzed parameters include BVP amplitude, heart rate (HR), and HRV components (LF, HF, LF/HF).

In their study, Brunzini et al. (2021b) collected a PPG signal to extract HR and inter-beat intervals (IBI), combining these with other physiological (EDA, eye data) and subjective (Numerical Analog Scale for stress and NASA-TLX for perceived workload) measurements to propose a comprehensive framework for assessing mental and physical load using portable sensors. In aviation, Wang et al. (2022) also used PPG to derive HRV as an indicator of MWL in pilots during flight simulation. In the construction sector, Shayesteh et al. (2023) combined PPG, EEG, and EDA with a deep neural network to evaluate human-robot collaboration: PPG+EDA predicted cognitive load with 60% accuracy, EEG alone reached 72%, and the combination of the three measures achieved 78% accuracy.

Thus, it can be retained that features extracted from physiological PPG signals (HR, HRV: IBI, pNN50/20) can be used to effectively assess mental workload in field settings.

### 3.12 Functional near-infrared spectroscopy (fNIRS)

Another measure of brain activity that has been validated for MWL assessment is functional near-infrared spectroscopy (fNIRS). This technique serves as a non-invasive and motion-tolerant brain imaging method (Afergan et al., 2014; Solovey et al., 2009). It utilizes near-infrared light to measure variations in cerebral blood oxygenation, where oxyhemoglobin (HbO_2_) converts to deoxyhemoglobin (HbR) during neuronal activity. By leveraging the principle of neurovascular coupling, which posits that active brain regions require increased blood flow due to higher metabolic demand, fNIRS provides an indirect assessment of brain activity.

One of the primary cortical regions studied for MWL using fNIRS is the prefrontal cortex. This area is associated with executive functions involved in cognitive processing related to MWL (Ayaz et al., 2012; Baddeley, 2012; Miller and Cohen, 2001). However, the literature highlights that photon absorption may be affected by hair, making fNIRS most reliable for regions such as the prefrontal cortex, although other areas (e.g., the parietal cortex) also play a role in workload assessment (Aricò et al., 2016).

fNIRS presents several advantages noted in the literature. It is safe, portable, minimally affected by movement artifacts, and does not require conductive gel or scalp abrasion. Unlike electroencephalography (EEG), it is less sensitive to electro-oculographic artifacts, environmental electrical noise, and facial muscle activity (Aricò et al., 2016; Durantin et al., 2014). Additionally, it offers a spatial resolution of ~1 cm^2^, superior to that of EEG, and is suitable for simultaneous use with EEG (Ayaz et al., 2012; Strangman et al., 2002).

The dependent variable used by Ayaz et al. (2012) is the average oxygenation change (HbO_2_-HbR) calculated using the Modified Beer-Lambert Law (MBLL). These authors demonstrated that fNIRS can be used in ecologically valid settings to assess the MWL of air traffic controllers (ATC). They observed a more pronounced increase in cerebral oxygenation in the anterior medial prefrontal cortex as workload increased, confirming that activation in these areas is a reliable measure of MWL in real-world conditions. Notably, their study suggested a decline in fNIRS measures with increased expertise and practice (e.g., piloting unmanned aerial vehicles), illustrating that brain activation in these frontal regions, linked to attentional and control processes, can serve as both an indicator of operator expertise and a reflection of neuroplasticity associated with training (Ayaz et al., 2012; Kelly and Garavan, 2005).

In an office task context, Midha et al. (2021) assessed MWL variation and found an increase in oxygenated hemoglobin and a decrease in deoxygenated hemoglobin during cognitively demanding tasks. Their findings indicate that MWL can be measured at the prefrontal level using fNIRS in office work conditions, aligning with subjective assessments. This technology has also been applied in the maritime sector to objectively assess MWL for safety purposes (e.g., reducing errors and accidents). For instance, Fan and Yang (2023) employed fNIRS to train a predictive model based on an artificial neural network (ANN) capable of identifying high mental load situations in seafarers. The dependent variables considered included HbO_2_, HbR, and total hemoglobin (Hb) recorded at the prefrontal cortex. Results indicated that these psychophysiological data could estimate MWL with 95% accuracy. However, it is important to note that a study has shown that fNIRS measures in air traffic control (ATC) tasks appeared to plateau, whereas subjective measures (Instantaneous Self-Assessment, ISA; Tattersall and Foord, 1996) continue to increase with task complexity (Harrison et al., 2014). This observation illustrates the complementarity of objective and subjective approaches in cognitive state assessment.

Durantin et al. (2014) emphasize that, contrary to the hypothesis that the autonomic nervous system (ANS) and the central nervous system (CNS) reach a saturation point when demands exceed available resources, cognitive resources follow a quadratic pattern similar to the inverted U-curve proposed by Yerkes and Dodson (1908). Thus, the performance decline following mental overload is attributed to reduced neuronal activity in the prefrontal regions, particularly in the dorsolateral prefrontal cortex (DLPFC). This work shows that it is possible to assess MWL both centrally and at the level of the ANS by probing neurophysiological activity. Indeed, as mentioned above, MWL exerts an influence on ANS activity, which can be measured through heart rate variability (HRV; Fallahi et al., 2016), pupil size (Tsai et al., 2007), electrodermal activity (Elena and Anastasia, 2021), respiration (Backs et al., 2000), biochemical markers (Murai, 2017), or temperature (Murai et al., 2008), all of which are regulated by the ANS and are involuntary reactions. MWL also affects the CNS, as demonstrated by neuronal activity measurements obtained via EEG or fNIRS (Midha et al., 2021; Saedi et al., 2022).

In summary, it can be retained that fNIRS is a reliable technique for assessing mental workload in field settings, with HbO_2_ and HbR emerging as key metrics of interest.

### 3.13 Electromyography (EMG)

Measurements of electrical muscle activity (EMG) are frequently associated with MWL. For instance, Fallahi et al. (2016) assessed the MWL of 16 road traffic control center operators during periods of rest, low traffic density, and high traffic density, combining physiological (HRV, EMG) and subjective (NASA-TLX) measures. Their findings indicate an increase in heart rate (HR), the LF/HF ratio, and the EMG amplitude of the trapezius muscle as traffic density increased. The increase in workload (both physical and mental) results in intensified muscle contraction (O'Donnell and Eggemeier, 1986).

Furthermore, the placement of EMG electrodes on the trapezius muscle (at the shoulder) is also used as an indicator of emotional stress (Cacioppo and Tassinary, 1990; Wijsman et al., 2010). Lastly, Hancock et al. (2021), in a review on MWL assessment methods, highlight that EMG measurements serve as a valuable tool for examining cognitive states related to stress, tension, and mental workload.

EMG amplitude at the trapezius muscle is a reliable and indicative measure of mental workload level in the workplace.

### 3.14 Speech analysis

Finally, some studies rely on speech analysis (loudness, fundamental frequency, speech rate, etc.) to detect signs of cognitive overload. However, this remains rare in MWL research, and there is still no clear consensus on the most robust vocal indicators. In the studied sample, only one research study (Cosić et al., 2019) used speech analysis to identify high MWL phases in air traffic controllers (ATC) during a simulation task. The authors combined several MWL assessment techniques and based their approach on the premise that voice analysis is considered a non-invasive method for measuring various cognitive states (stress, fatigue, MWL; Greeley et al., 2006; Whitmore and Fisher, 1996).

Speech production—being a complex process that mobilizes both the central nervous system and the peripheral nervous system—the latter depending notably on the autonomic nervous system (ANS)—vocal modifications can reflect variations in sympathetic activity. Cosić et al. (2019) emphasize that cognitive overload can be understood as a form of stress, aligning with the definition proposed by Murray et al. (1996, p. 5): “Stress is a psycho-physiological state characterized by subjective strain, dysfunctional physiological activity and deterioration of performance.” In other words, MWL constitutes an additional demand imposed on the cognitive system, reinforcing the idea that a stressful context often results in alterations in vocal production (Patil et al., 2013; Womack and Hansen, 1999).

#### 3.14.1 Vocal markers of sympathetic activity

Various speech characteristics, such as vocal intensity (mean, median), spectral parameters (FO = fundamental frequency; MFCC = Mel Frequency Cepstral Coefficients, jitter, etc.), or speech rate (number/mean of voice segments per second), are used to describe the speech signal. Spectral and autocorrelation analyses are employed to assess indices of sympathetic activity.

In the literature, fundamental frequency (FO) and vocal intensity (loudness) are particularly associated with sympathetic activity, increasing as sympathetic activation intensifies (Cosić et al., 2016). Similarly, an increase in cognitive load has already been linked to a increase in speech rate and mean FO (Scherer et al., 2002), with similar results observed for stress (Giddens et al., 2013; Lu et al., 2012; Wittels et al., 2002).

#### 3.14.2 The loudness mean difference (LMD) indicator

In their study, Cosić et al. (2019) extracted 26 vocal characteristics and focused on the difference in mean vocal intensity (LMD = loudness mean during a high-load phase – loudness mean during a low-load phase). According to them, a low LMD reflects lower sympathetic activity and a better ANS balance. Consequently, individuals exhibiting this characteristic may be better suited for demanding jobs, such as piloting or air traffic control. In summary, it appears that speech features (voice fundamental frequency, loudness, speech rate) are effective for assessing mental workload in field conditions.

## 4 Discussion

The main objective of this review was to identify studies conducted in the field or under field-like conditions that assess mental workload (MWL) using neurophysiological measures. The underlying goal was to determine the most reliable and valid indicators for objectively estimating MWL in real professional contexts. Indeed, neurophysiological measurements have the advantage of not interrupting workers during task execution and overcoming the limitations inherent to delayed self-assessments (subjective biases, inaccuracies, etc.), with subjective measures (e.g., NASA-TLX) remaining widely used.

One of the initial concerns was the actual use of these measurement methods in real-world settings, particularly considering certain constraints: acquisition costs, complex data analysis, relative intrusiveness, etc. However, results indicate that these techniques are effectively implemented in professional environments and are becoming increasingly accessible with technological advancements, such as miniaturization, smartwatches, and wearable sensors.

Numerous studies highlight the relevance of cardiovascular indicators, particularly electrocardiogram (ECG) and heart rate (HR), which are relatively low-cost and minimally intrusive. When combined with subjective scales (e.g., NASA-TLX), they constitute robust tools for MWL assessment in professional settings (Jung and Jung, 2001). The review also highlights other techniques:

- **Monitoring of ocular activity** (pupil diameter, blinks, fixations, etc.),
- **Measurements of brain activity** (EEG, fNIRS),
- **Electrodermal activity (EDA)**,
- **Respiration** (frequency, amplitude),
- **Hormonal indicators** (cortisol, salivary amylase),
- **Skin temperature** (facial region, nose),
- **Blood volume pulse (BVP) measured by photoplethysmography**,
- **Electrical muscle activity (EMG)**,
- **Speech analysis**.

In general, these different approaches are frequently combined with subjective scales (e.g., NASA-TLX, ISA) to leverage the complementarity between objective measurements and workers' self-reported perceptions. Table 2 illustrates the various techniques used across the different work domains represented in the review and that can be applied in the field. Overall, it appears that cardiac activity measures, eye-tracking, and brain activity measurement techniques account for nearly half of the assessment methods used. For field evaluation, it would be appropriate to use one or more of these techniques in combination with subjective measures. Studies emphasize that certain physiological metrics may plateau as MWL increases, whereas subjective measures continue to evolve (Harrison et al., 2014). There is no “perfect measure” of mental workload: each indicator (ECG, EEG, fNIRS, EDA, etc.) has its own advantages and limitations (intrusiveness, sensitivity to environmental factors, cost, etc.). Studies converge on the necessity of a multimodal approach, integrating multiple physiological measurements and subjective self-assessments to achieve a more accurate estimation of actual MWL (Charles and Nixon, 2019; Lehrer et al., 2010; Sriranga et al., 2023; Hancock et al., 1985).

### 4.1 Key indicators

The reviewed studies highlight specific physiological indicators that are particularly sensitive and consistently associated with MWL in field settings. Among them, ECG, eye data, and brain activity measures (EEG and fNIRS) together represent over half of the techniques employed in the reviewed literature. These modalities appear especially promising for assessing MWL in real-world environments.

For ECG, reliable metrics include heart rate (HR) and heart rate variability (HRV) indices such as mean RRI, LF/HF, RMSSD, pNN50, and SDNN, as well as non-linear indicators like Shannon entropy (Cinaz et al., 2013; Fallahi et al., 2016). These metrics are frequently used and have demonstrated robustness in detecting MWL variations.

Fo eye data, several studies consistently report that pupil diameter, blink rate, and fixation frequency and duration are sensitive to changes in MWL (Naik et al., 2022; Das et al., 2020). These indicators can be complemented by entropy-based gaze measures, which have also shown promising results in recent studies.

For EEG, spectral power in the theta and beta bands over the frontal cortex, alpha power over parietal regions, and the EEG workload index are repeatedly identified as reliable neurometrics for MWL assessment in real-world tasks (Aricò et al., 2015; Kosti et al., 2018; Saedi et al., 2022).

Regarding electrodermal activity (EDA), metrics such as average SC amplitude, mean SCL, and SCR peak amplitude have demonstrated consistent associations with MWL (Elena and Anastasia, 2021; Lagomarsino et al., 2022). Similarly, respiratory rate and breathing depth are valuable indicators for monitoring MWL at the workplace (Brunzini et al., 2021b; Roscoe, 1992).

Although hormonal markers such as salivary alpha-amylase and cortisol have been explored (Cinaz et al., 2013; Murai, 2017), the latter presents mixed findings and is more susceptible to circadian and emotional variability. In contrast, salivary NO3 and alpha-amylase have emerged as more promising for field assessment.

Skin temperature variation, particularly nasal temperature, may also serve as a reliable MWL indicator, especially when combined with other measures (Murai et al., 2008).

From photoplethysmography (PPG), extracted features such as inter-beat interval (IBI) and pNN50 can effectively reflect MWL levels. EMG amplitude at the trapezius muscle is another robust indicator, particularly under combined physical and cognitive demands.

For brain hemodynamic activity, fNIRS has proven to be a reliable technique, with HbO_2_ and HbR identified as relevant metrics (Ayaz et al., 2012). Lastly, speech features, including fundamental frequency, loudness, and speech rate, are effective in evaluating MWL in field conditions (Cosić et al., 2019).

To help guide researchers and field practitioners in selecting appropriate methods, Table 2 summarizes which techniques were most frequently applied across different work domains in the reviewed studies. This table can support informed decisions when choosing the relevant physiological metrics for MWL assessment in operational settings.

It is also relevant to compare the findings from this review with those of previous reviews that included both laboratory and field studies. Many of the physiological techniques and metrics identified here, such as heart rate variability (HRV), EEG spectral power in theta and beta bands, electrodermal activity (EDA), and oculomotor indicators, have also been reported as effective for MWL assessment in prior systematic reviews that covered both laboratory and field-based studies (Charles and Nixon, 2019).

However, Charles and Nixon (2019) emphasize the inherent limitations in generalizing laboratory data to field applications. For example, Wilson (1992) observed that heart rate variations can reach up to 50% in real-world settings, compared to only 10% in the lab. Similarly, earlier studies have shown that correlations between lab and field physiological data tend to be weak (Johnston et al., 1990). This discrepancy highlights the impact of contextual complexity in operational environments and the necessity of distinguishing between simulated and real tasks.

Therefore, this review offers a complementary perspective by focusing solely on field-based studies. It provides a targeted synthesis of physiological techniques that have demonstrated reliability and applicability in real-world working conditions, thus helping bridge the gap between experimental research and operational practice.

### 4.2 Links between MWL and stress

This review highlights that many physiological metrics used to assess MWL are also applied to stress evaluation. Some authors even consider cognitive overload as a form of stress (Cosić et al., 2019). Several studies suggest that an excessive MWL level negatively impacts performance, increases the risk of errors and psychological disorders (burnout, emotional distress), and adversely affects workers' health (Leslie and Hutchinson, 2018; Qu, 2013; Das et al., 2020; Han et al., 2017; Rusnock and Borghetti, 2018).

MWL and stress appear to share partially overlapping neurophysiological mechanisms and physiological markers. Similar variations in the sympathovagal ratio (LF/HF), pupil size, brain activity (decrease in alpha, increase in beta), and skin temperature have been observed in both stressful situations and high mental workload conditions (Alberdi et al., 2016; Tuscan et al., 2013). Cosić et al. (2019) suggests that the physiological consequences of MWL on speech features are comparable to those observed in stressful situations, particularly through the overactivation of the sympathetic branch of the ANS. MWL and stress exert similar effects on the autonomic nervous system, and several studies acknowledge that excessive cognitive load can induce stress in workers (Cinaz et al., 2013).

However, the literature emphasizes the need to clearly distinguish MWL from stress. Some studies have demonstrated distinct vocal profiles depending on whether it is “psychological stress” or high cognitive load (Scherer et al., 2002). Indeed, stress can occur even with a low MWL, while a highly demanding task does not necessarily trigger significant stress (Gaillard, 1993; Causse et al., 2022). It is therefore essential to closely examine the neurophysiological substrates and the distinct (or shared) mechanisms underlying each of these states.

Alsuraykh et al. (2019) addressed this issue and demonstrated that stress and MWL are highly interconnected. They further note, as previously mentioned, that their respective measures influence one another. In fact, the authors highlight similarities between Cox (1978) transactional stress theory and Wickens (2008) framework for MWL. Causse et al. (2022, p. 1012) observe that MWL and stress are often indistinctly referred to in the literature under the term “mental stress.”

The prefrontal cortex (PFC) plays a central role: it is both a key component of executive control (and thus working memory, which is highly engaged under increased MWL) and is involved in stress regulation (through modulation of amygdala and locus coeruleus responses; Bremner, 2006; Pozzi and Matteoli, 2018; Dehais et al., 2020). Increased MWL leads to the recruitment of the lateral frontoparietal network, while stress induces increased ECN (executive control network) activity (Causse et al., 2022). Similar to stress, increased MWL also deactivates the PFC (Dehais et al., 2020). This suggests that MWL and stress share common functional structures at the brain level, which is reflected in their measurement through overlapping physiological metrics.

Furthermore, it has been shown that dopamine (DA) mediates the cognitive effort exerted in task execution (Westbrook and Braver, 2016). When its release increases, PFC deactivation is observed, similar to what occurs following a stressful situation. The PFC operates under the quadratic influence of neuromodulators (dopamine, noradrenaline), which exhibit an inverted U-shaped relationship with these neurons (Dehais et al., 2020). These key neuromediators may thus be involved in both stress and MWL situations (Arnsten, 2009; Dehais et al., 2020). These studies demonstrate the interrelations between these two cognitive states.

It remains necessary to clarify the operationalization of MWL and stress in measurement protocols and to consider explicitly assessing the stress component using additional dedicated indicators or questionnaires during MWL evaluation. To do so, researchers should clearly define in advance whether the goal is to assess MWL, stress, or both. When the primary focus is on MWL, efforts should be made to minimize the induction of stress during experimental tasks. However, given the conceptual and physiological overlap between the two constructs, it may be necessary, when stress cannot be excluded, to evaluate its contribution. This can be achieved by combining physiological indicators with validated stress questionnaires such as the STAI (State-Trait Anxiety Inventory), particularly when MWL is manipulated experimentally. This approach, already recommended in the literature (Alsuraykh et al., 2019), would help to better disentangle the respective contributions of MWL and stress in applied settings.

### 4.3 Limitations of the review and future directions

This review has certain limitations, including its restriction to three databases and the use of specific keywords (which may have excluded some work). In particular, the combined use of the terms “*mental”* and “*cognitive workload”* within a single search query could have significantly limited the number of retrieved results. Nevertheless, it offers a substantial overview of the published research and proposes a synthesis integrating the variety of physiological measures studied. In the future, a more in-depth analysis could consist of comparing the predictive validity of the different metrics (ECG, EEG, EDA, etc.) to discriminate various levels of MWL, taking into account the level of stress and inter-individual differences (experience, age, etc.).

## 5 Conclusion

Mental workload (MWL) is a critical concern for employee health, safety, and performance across a wide range of sectors, including industry, transportation, and healthcare. This systematic review highlights the potential of physiological measures, such as cardiac, cerebral, electrodermal, respiratory, and oculomotor signals, for assessing MWL directly in real-world settings, thus offering a valuable complement to traditional subjective approaches.

Recent progress in wearable technologies and portable sensors has significantly enhanced the feasibility of real-time physiological monitoring in the workplace. In particular, emerging studies employing predictive modeling techniques, such as artificial intelligence and classification algorithms, have demonstrated that it is possible to detect and predict states of cognitive underload and overload from physiological signals like EEG, EDA, and PPG (Zhang et al., 2020; Shayesteh et al., 2023; Liu et al., 2021). When coupled with neurophysiological data, these methods show strong potential for implementing adaptive systems capable of monitoring MWL continuously and preventing human error in high-risk environments such as nuclear power plants or construction sites.

From a prevention perspective, however, it remains essential to combine these objective measures with subjective assessments and performance-based indicators. This multimodal approach allows for a more comprehensive understanding of mental demands by accounting not only for the physiological cost of tasks but also for individual perception and environmental context.

Finally, future research should prioritize the simultaneous integration of multiple physiological measures, the development of standardized *in situ* protocols, and a deeper investigation into the relationship between MWL and stress, two constructs that, while closely linked, must be clearly distinguished in operational evaluations.

## Data Availability

The original contributions presented in the study are included in the article/supplementary material, further inquiries can be directed to the corresponding authors.

## References

[B1] Afergan D. Peck E. M. Solovey E. T. Jenkins A. Hincks S. W. Brown E. T. . (2014). “Dynamic difficulty using brain metrics of workload,” in Proceedings of the SIGCHI Conference on Human Factors in Computing Systems, Toronto, Ontario, Canada (New York, NY: Association for Computing Machinery). 10.1145/2556288.2557230

[B2] Alberdi A. Aztiria A. Basarab A. (2016). Towards an automatic early stress recognition system for office environments based on multimodal measurements: a review. J. Biomed. Inform. 59, 49–75. 10.1016/j.jbi.2015.11.007 26621099

[B3] Allsop J. Gray R. (2014). Flying under pressure: effects of anxiety on attention and gaze behavior in aviation. J. Appl. Res. Memory Cogn. 3, 63–71. 10.1016/j.jarmac.2014.04.010

[B4] Alsuraykh N. H. Wilson M. L. Tennent P. Sharples S. (2019). “How stress and mental workload are connected,” in Proceedings of the 13th EAI International Conference on Pervasive Computing Technologies for Healthcare, Trento, Italy (New York, NY: Association for Computing Machinery). 10.1145/3329189.3329235

[B5] Antonenko P. Paas F. Grabner R. van Gog T. (2010). Using electroencephalography to measure cognitive load. Educ. Psychol. Rev. 22, 425–438. 10.1007/s10648-010-9130-y

[B6] Arico P. Borghini G. Di Flumeri G. Bonelli S. Golfetti A. Graziani I. . (2017). Human factors and neurophysiological metrics in air traffic control: a critical review. IEEE Rev. Biomed. Eng. 10, 250–263. 10.1109/RBME.2017.2694142 28422665

[B7] Aricò P. Borghini G. Di Flumeri G. Colosimo A. Pozzi S. Babiloni F. (2016). “Chapter 10 - A passive brain–computer interface application for the mental workload assessment on professional air traffic controllers during realistic air traffic control tasks,” in Progress in Brain Research, Vol. 228, ed. Coyle D. (Elsevier), pp. 295–328. 10.1016/bs.pbr.2016.04.021 27590973

[B8] Aricò P. Borghini G. Flumeri G. D. Colosimo A. Graziani I. Imbert J.-P. . (2015). “Reliability over time of EEG-based mental workload evaluation during air traffic management (ATM) tasks,” in 2015 37th Annual International Conference of the IEEE Engineering in Medicine and Biology Society (EMBC) (Milan: IEEE). 10.1109/EMBC.2015.7320063 26737963

[B9] Aricò P. Borghini G. Graziani I. Taya F. Sun Y. Bezerianos A. . (2014). Towards a multimodal bioelectrical framework for the online mental workload evaluation. Annu. Int. Conf. IEEE Eng. Med. Biol. Soc. 2014, 3001–3004. 10.1109/EMBC.2014.6944254 25570622

[B10] Arnsten A. F. T. (2009). Stress signalling pathways that impair prefrontal cortex structure and function. Nat. Rev. Neurosci. 10, 410–422. 10.1038/nrn2648 19455173 PMC2907136

[B11] Aston-Jones G. Cohen J. D. (2005). An integrative theory of locus coeruleus-norepinephrine function: adaptive gain and optimal performance. Annu. Rev. Neurosci. 28, 403–450. 10.1146/annurev.neuro.28.061604.135709 16022602

[B12] Ayaz H. Shewokis P. A. Bunce S. Izzetoglu K. Willems B. Onaral B. (2012). Optical brain monitoring for operator training and mental workload assessment. NeuroImage 59, 36–47. 10.1016/j.neuroimage.2011.06.023 21722738

[B13] Backs R. W. Navidzadeh H. T. Xu X. (2000). Cardiorespiratory indices of mental workload during simulated air traffic control. Proc. Hum. Fact. Ergon. Soc. Annu. Meet. 44, 89–92. 10.1177/154193120004401323

[B14] Baddeley A. (2012). Working memory: theories, models, and controversies. Annu. Rev. Psychol. 63, 1–29. 10.1146/annurev-psych-120710-100422 21961947

[B15] Barreto A. Zhai J. Rishe N. Gao Y. (2007). “Significance of pupil diameter measurements for the assessment of affective state in computer users,” in Advances and Innovations in Systems, Computing Sciences and Software Engineering, ed. Elleithy K. (Dordrecht: Springer), 59–64.

[B16] Bednarik R. Koskinen J. Vrzakova H. Bartczak P. Elomaa A. P. (2018). “Blink-based estimation of suturing task workload and expertise in microsurgery,” in 2018 IEEE 31st International Symposium on Computer-Based Medical Systems (CBMS) (Karlstad: IEEE). 10.1109/CBMS.2018.00048

[B17] Borghini G. Aricò P. Graziani I. Salinari S. Sun Y. Taya F. . (2016). Quantitative assessment of the training improvement in a motor-cognitive task by using EEG, ECG and EOG signals. Brain Topogr. 29, 149–161. 10.1007/s10548-015-0425-7 25609212

[B18] Bremner J. D. (2006). Traumatic stress: effects on the brain. Dial. Clin. Neurosci. 8, 445–461. 10.31887/DCNS.2006.8.4/jbremner 17290802 PMC3181836

[B19] Brookings J. B. Wilson G. F. Swain C. R. (1996). Psychophysiological responses to changes in workload during simulated air traffic control. Biol. Psychol. 42, 361–377. 10.1016/0301-0511(95)05167-8 8652753

[B20] Brunzini A. Grandi F. Peruzzini M. Pellicciari M. (2021a). Virtual training for assembly tasks: a framework for the analysis of the cognitive impact on operators. Procedia Manufactur. 55, 527–534. 10.1016/j.promfg.2021.10.072

[B21] Brunzini A. Peruzzini M. Grandi F. Khamaisi R. K. Pellicciari M. (2021b). A preliminary experimental study on the workers' workload assessment to design industrial products and processes. Appl. Sci. 11:12066. 10.3390/app112412066

[B22] Byrne A. (2013). Mental workload as a key factor in clinical decision making. Adv. Health Sci. Educ. 18, 537–545. 10.1007/s10459-012-9360-5 22411354

[B23] Cacioppo J. T. Tassinary L. G. (1990). Inferring psychological significance from physiological signals. Am. Psychol. 45, 16–28. 10.1037//0003-066X.45.1.16 2297166

[B24] Caramia F. Angeletti P. U. Levi-Montalcini R. (1962). Experimental analysis of the mouse submaxillary salivary gland in relationship to its nerve-growth factor content. Endocrinology 70, 915–922. 10.1210/endo-70-6-915 13876428

[B25] Castaldo R. Melillo P. Bracale U. Caserta M. Triassi M. Pecchia L. (2015). Acute mental stress assessment via short term HRV analysis in healthy adults: a systematic review with meta-analysis. Biomed. Signal Process. Control 18, 370–377. 10.1016/j.bspc.2015.02.012

[B26] Causse M. Lepron E. Mandrick K. Peysakhovich V. Berry I. Callan D. . (2022). Facing successfully high mental workload and stressors: an fMRI study. Hum. Brain Mapp. 43, 1011–1031. 10.1002/hbm.25703 34738280 PMC8764488

[B27] Causse M. Sénard J. M. Démonet J. F. Pastor J. (2010). Monitoring cognitive and emotional processes through pupil and cardiac response during dynamic versus logical task. Appl. Psychophysiol. Biofeedback 35, 115–123. 10.1007/s10484-009-9115-0 19816770

[B28] Charles R. L. Nixon J. (2019). Measuring mental workload using physiological measures: a systematic review. Appl. Ergon. 74, 221–232. 10.1016/j.apergo.2018.08.028 30487103

[B29] Chen J. Song X. Lin Z. (2016). Revealing the “Invisible Gorilla” in construction: estimating construction safety through mental workload assessment. Automat. Construct. 63, 173–183. 10.1016/j.autcon.2015.12.018

[B30] Chida Y. Steptoe A. (2009). Cortisol awakening response and psychosocial factors: a systematic review and meta-analysis. Biol. Psychol. 80, 265–278. 10.1016/j.biopsycho.2008.10.004 19022335

[B31] Chrousos G. P. (2009). Stress and disorders of the stress system. Nat. Rev. Endocrinol. 5, 374–381. 10.1038/nrendo.2009.106 19488073

[B32] Cinaz B. Arnrich B. La Marca R. Tröster G. (2013). Monitoring of mental workload levels during an everyday life office-work scenario. Personal Ubiquitous Comput. 17, 229–239. 10.1007/s00779-011-0466-1

[B33] Cohen Y. Golan M. Singer G. Faccio M. (2018). Workstation–operator interaction in 4.0 era: WOI 4.0. IFAC-PapersOnLine 51, 399–404. 10.1016/j.ifacol.2018.08.327

[B34] Coronado E. Kiyokawa T. Ricardez G. A. G. Ramirez-Alpizar I. G. Venture G. Yamanobe N. (2022). Evaluating quality in human-robot interaction: a systematic search and classification of performance and human-centered factors, measures and metrics towards an industry 5.0. J. Manufactur. Syst. 63, 392–410. 10.1016/j.jmsy.2022.04.007

[B35] Cosić K. Popović S. Kukolja D. Dropuljić B. Ivanec D. Tonković M. (2016). Multimodal analysis of startle type responses. Comput. Methods Progr. Biomed. 129, 186–202. 10.1016/j.cmpb.2016.01.002 26826902

[B36] Cosić K. Popović S. Šarlija M. Mijić I. Kokot M. KesedŽić I. . (2019). New tools and methods in selection of air traffic controllers based on multimodal psychophysiological measurements. IEEE Access 7, 174873–174888. 10.1109/ACCESS.2019.2957357

[B37] Cox T. (1978). Stress. London: Macmillan Press. Available online at: https://books.google.fr/books?id=43YdAQAAMAAJ (accessed April 18, 2023).

[B38] Das S. Maiti J. Krishna O. B. (2020). Assessing mental workload in virtual reality based EOT crane operations: a multi-measure approach. Int. J. Indus. Ergon. 80:103017. 10.1016/j.ergon.2020.103017

[B39] Dehais F. Lafont A. Roy R. Fairclough S. (2020). A neuroergonomics approach to mental workload, engagement and human performance. Front. Neurosci. 14:268. 10.3389/fnins.2020.00268 32317914 PMC7154497

[B40] Di Nocera F. Camilli M. Terenzi M. (2007). A random glance at the flight deck: Pilots' scanning strategies and the real-time assessment of mental workload. J. Cogn. Eng. Decision Making 1, 271–285. 10.1518/155534307X255627

[B41] Di Stasi L. L. Diaz-Piedra C. Rieiro H. Sánchez Carrión J. M. Martin Berrido M. Olivares G. . (2016). Gaze entropy reflects surgical task load. Surg. Endosc. 30, 5034–5043. 10.1007/s00464-016-4851-8 26983440

[B42] Dias R. D. Zenati M. A. Stevens R. Gabany J. M. Yule S. J. (2019). Physiological synchronization and entropy as measures of team cognitive load. J. Biomed. Inform. 96:103250. 10.1016/j.jbi.2019.103250 31295623 PMC7226673

[B43] Dickerson S. S. Kemeny M. E. (2004). Acute stressors and cortisol responses: a theoretical integration and synthesis of laboratory research. Psychol. Bull. 130, 355–391. 10.1037/0033-2909.130.3.355 15122924

[B44] Durantin G. Gagnon J. F. Tremblay S. Dehais F. (2014). Using near infrared spectroscopy and heart rate variability to detect mental overload. Behav. Brain Res. 259, 16–23. 10.1016/j.bbr.2013.10.042 24184083

[B45] Elena A. K. Anastasia V. K. (2021). “Skin conductance as a real-time indicator of the high/low workload during flight simulator sessions (case study),” in 2021 International Conference on Cyberworlds (CW) (Caen: IEEE).

[B46] Fairclough S. H. Venables L. Tattersall A. (2005). The influence of task demand and learning on the psychophysiological response. Int. J. Psychophysiol. 56, 171–184. 10.1016/j.ijpsycho.2004.11.003 15804451

[B47] Fallahi M. Motamedzade M. Heidarimoghadam R. Soltanian A. R. Miyake S. (2016). Effects of mental workload on physiological and subjective responses during traffic density monitoring: a field study. Appl. Ergon. 52, 95–103. 10.1016/j.apergo.2015.07.009 26360199

[B48] Fan S. Yang Z. (2023). Towards objective human performance measurement for maritime safety: a new psychophysiological data-driven machine learning method. Reliabil. Eng. Syst. Saf. 233:109103. 10.1016/j.ress.2023.109103

[B49] Fan X. Zhao C. Hu H. Jiang Y. (2020). “Review of the evaluation methods of mental workload,” in Advances in Physical Ergonomics and Human Factors, eds. Goonetilleke R. S. Karwowski W. (Cham: Springer International Publishing), 165–172. 10.1007/978-3-030-20142-5_17

[B50] Gaillard A. W. K. (1993). Comparing the concepts of mental load and stress. Ergonomics 36, 991–1005. 10.1080/00140139308967972 8404841

[B51] Gao Q. Wang Y. Song F. Li Z. Dong X. (2013). Mental workload measurement for emergency operating procedures in digital nuclear power plants. Ergonomics 56, 1070–1085. 10.1080/00140139.2013.790483 23654299

[B52] Giddens C. L. Barron K. W. Byrd-Craven J. Clark K. F. Winter A. S. (2013). Vocal indices of stress: a review. J. Voice 27, 390.e321–390.e329. 10.1016/j.jvoice.2012.12.010 23462686

[B53] Grandjean E. (1980). Fitting the Task to the Man: An Ergonomic Approach. Taylor and Francis. Available online at: https://books.google.fr/books?id=bltRAAAAMAAJ (accessed April 21, 2023).

[B54] Greeley H. P. Friets E. M. Wilson J. P. Raghavan S. Picone J. W. Berg J. (2006). “Detecting fatigue from voice using speech recognition,” in 2006 IEEE International Symposium on Signal Processing and Information Technology (Vancouver, BC: IEEE), 567–571. 10.1109/ISSPIT.2006.270865

[B55] Green M. S. Luz Y. Jucha E. Cocos M. Rosenberg N. (1986). Factors affecting ambulatory heart rate in industrial workers†. Ergonomics 29, 1017–1027. 10.1080/00140138608967215 3758021

[B56] Grobe S. Kakar R. S. Smith M. L. Mehta R. Baghurst T. Boolani A. (2017). Impact of cognitive fatigue on gait and sway among older adults: a literature review. Prev. Med. Rep. 6, 88–93. 10.1016/j.pmedr.2017.02.016 28271026 PMC5338901

[B57] Han L. Zhang Q. Chen X. Zhan Q. Yang T. Zhao Z. (2017). Detecting work-related stress with a wearable device. Comput. Indus. 90, 42–49. 10.1016/j.compind.2017.05.004

[B58] Hancock G. M. Longo L. Young M. S. Hancock P. A. (2021). “Mental workload,” in Handbook of Human Factors and Ergonomics, eds. Salvendy G. Karwowski W. (John Wiley & Sons, Inc.), 203–226. 10.1002/9781119636113.ch7

[B59] Hancock P. A. Meshkati N. Robertson M. M. (1985). Physiological reflections of mental workload. Aviat. Space Environ. Med. 56, 1110–1114. 3907615

[B60] Harrison J. Izzetoglu K. Ayaz H. Willems B. Hah S. Ahlstrom U. . (2014). Cognitive workload and learning assessment during the implementation of a next-generation air traffic control technology using functional near-infrared spectroscopy. IEEE Trans. Human Machine Syst. 44, 429–440. 10.1109/THMS.2014.2319822

[B61] Hart S. G. Staveland L. E. (1988). “Development of NASA-TLX (task load index): results of empirical and theoretical research,” in Advances in Psychology, Vol. 52, eds. Hancock P. A. Meshkati N. (North-Holland), 139–183. 10.1016/S0166-4115(08)62386-9

[B62] Heard J. Harriott C. E. Adams J. A. (2018). A survey of workload assessment algorithms. IEEE Trans. Human Machine Syst. 48, 434–451. 10.1109/THMS.2017.2782483

[B63] Hermans E. J. Henckens M. J. A. G. Joëls M. Fernández G. (2014). Dynamic adaptation of large-scale brain networks in response to acute stressors. Trends Neurosci. 37, 304–314. 10.1016/j.tins.2014.03.006 24766931

[B64] Iqbal M. U. Srinivasan B. Srinivasan R. (2020). Dynamic assessment of control room operator's cognitive workload using electroencephalography (EEG). Comput. Chem. Eng. 141:106726. 10.1016/j.compchemeng.2020.106726

[B65] Jensen O. Tesche C. D. (2002). Frontal theta activity in humans increases with memory load in a working memory task. Euro. J. Neurosci. 15, 1395–1399. 10.1046/j.1460-9568.2002.01975.x 11994134

[B66] Johnston D. W. Anastasiades P. Wood C. (1990). The relationship between cardiovascular responses in the laboratory and in the field. Psychophysiology 27, 34–44. 10.1111/j.1469-8986.1990.tb02175.x 2339186

[B67] Jung H. S. Jung H.-S. (2001). Establishment of overall workload assessment technique for various tasks and workplaces. Int. J. Indus. Ergon. 28, 341–353. 10.1016/S0169-8141(01)00040-3

[B68] Kabilmiharbi N. Kamaliana Khamis N. Azila Noh N. (2022). Commonly used assessment method to evaluate mental workload for multiple driving distractions: a systematic review. Iran. J. Public Health 51, 482–494. 10.18502/ijph.v51i3.8924 35865045 PMC9276604

[B69] Kaklauskas A. Zavadskas E. K. Seniut M. Dzemyda G. Stankevic V. Simkevičius C. . (2011). Web-based biometric computer mouse advisory system to analyze a user's emotions and work productivity. Eng. Appl. Artif. Intell. 24, 928–945. 10.1016/j.engappai.2011.04.006

[B70] Kelly A. M. Garavan H. (2005). Human functional neuroimaging of brain changes associated with practice. Cereb. Cortex 15, 1089–1102. 10.1093/cercor/bhi005 15616134

[B71] Khaksari K. Condy E. Millerhagen J. B. Anderson A. A. Dashtestani H. Gandjbakhche A. H. (2019). Effects of performance and task duration on mental workload during working memory task. Photonics 6:94. 10.3390/photonics6030094

[B72] Kitamura K. Murai K. Wakida S.-i. (2016). “Evaluation of mental workload of sea pilot and captain using salivary NO3,” in 2016 World Automation Congress (WAC) (Rio Grande). 10.1109/WAC.2016.7582972

[B73] Kohlmorgen J. Dornhege G. Braun M. Blankertz B. Müller K.-R. Curio G. . (2007). Improving Human Performance in a Real Operating Environment Through Real-Time Mental Workload Detection. Available online at: https://publica.fraunhofer.de/handle/publica/214953 (accessed March 15, 2023).

[B74] Kosti M. V. Georgiadis K. Adamos D. A. Laskaris N. Spinellis D. Angelis L. (2018). Towards an affordable brain computer interface for the assessment of programmers' mental workload. Int. J. Human Comput. Stud. 115, 52–66. 10.1016/j.ijhcs.2018.03.002

[B75] Kumar N. Lee S. C. (2022). Human-machine interface in smart factory: a systematic literature review. Technol. Forecast. Soc. Change 174:121284. 10.1016/j.techfore.2021.121284

[B76] Kuo Y.-C. J. Chen K.-H. S. (2022). “Chapter 2 - Electrophysiological assessment of respiratory function,” in Handbook of Clinical Neurology, Vol. 189, eds. Chen R. Guyenet P. G. (Amsterdam: Elsevier), 15–40. 10.1016/B978-0-323-91532-8.00002-1 36031302

[B77] Kurniawan H. Maslov A. V. Pechenizkiy M. (2013). “Stress detection from speech and Galvanic Skin Response signals,” in Proceedings of the 26th IEEE International Symposium on Computer-Based Medical Systems (Porto: IEEE). 10.1109/CBMS.2013.6627790

[B78] Laeng B. Sirois S. Gredebäck G. (2012). Pupillometry: a window to the preconscious? Perspect. Psychol. Sci. 7, 18–27. 10.1177/1745691611427305 26168419

[B79] Lagomarsino M. Lorenzini M. E Ajoudani A. (2022). An online framework for cognitive load assessment in industrial tasks. Robot. Comput. Integr. Manufactur. 78:102380. 10.1016/j.rcim.2022.102380

[B80] Lehrer P. Karavidas M. Lu S.-E. Vaschillo E. Vaschillo B. Cheng A. (2010). Cardiac data increase association between self-report and both expert ratings of task load and task performance in flight simulator tasks: an exploratory study. Int. J. Psychophysiol. 76, 80–87. 10.1016/j.ijpsycho.2010.02.006 20172000

[B81] Leslie C. Hutchinson A. D. (2018). Emotional distress when studying sensitive topics in psychology, and its relationship with hardiness and mental health. Higher Educ. Res. Dev. 37, 549–564. 10.1080/07294360.2018.1436525

[B82] Lim C. L. Rennie C. Barry R. J. Bahramali H. Lazzaro I. Manor B. . (1997). Decomposing skin conductance into tonic and phasic components. International Journal of Psychophysiol. 25, 97–109. 10.1016/S0167-8760(96)00713-1 9101335

[B83] Liu Y. Habibnezhad M. Jebelli H. (2021). Brainwave-driven human-robot collaboration in construction. Automat. Construct. 124:103556. 10.1016/j.autcon.2021.103556

[B84] Lu H. Frauendorfer D. Rabbi M. Mast M. S. Chittaranjan G. T. Campbell A. T. . (2012). “StressSense: detecting stress in unconstrained acoustic environments using smartphones,” in Proceedings of the 2012 ACM Conference on Ubiquitous Computing, Pittsburgh, Pennsylvania (New York, NY: Association for Computing Machinery). 10.1145/2370216.2370270

[B85] Mack A. (2003). Inattentional blindness: looking without seeing. Curr. Direct. Psychol. Sci. 12, 180–184. 10.1111/1467-8721.01256

[B86] Marinescu A. C. Sharples S. Ritchie A. C. Sánchez López T. McDowell M. Morvan H. P. (2018). Physiological parameter response to variation of mental workload. Hum. Fact. 60, 31–56. 10.1177/0018720817733101 28965433 PMC5777546

[B87] Mazur L. M. Mosaly P. R. Hoyle L. M. Jones E. L. Chera B. S. Marks L. B. (2014). Relating physician's workload with errors during radiation therapy planning. Prac. Radiat. Oncol. 4, 71–75. 10.1016/j.prro.2013.05.010 24890346

[B88] McFarland R. A. (1985). Relationship of skin temperature changes to the emotions accompanying music. Biofeedback Self Regul. 10, 255–267. 10.1007/BF00999346 3835976

[B89] Mehta R. K. (2016). Integrating physical and cognitive ergonomics. IIE Trans. Occup. Ergon. Hum. Fact. 4, 83–87. 10.1080/21577323.2016.1207475

[B90] Midha S. Maior H. A. Wilson M. L. Sharples S. (2021). Measuring mental workload variations in office work tasks using fNIRS. Int. J. Hum. Comput. Stud. 147:102580. 10.1016/j.ijhcs.2020.102580

[B91] Miller E. K. Cohen J. D. (2001). An integrative theory of prefrontal cortex function. Annu. Rev. Neurosci. 24, 167–202. 10.1146/annurev.neuro.24.1.167 11283309

[B92] Muldner K. Burleson W. (2015). Utilizing sensor data to model students' creativity in a digital environment. Comput. Hum. Behav. 42, 127–137. 10.1016/j.chb.2013.10.060

[B93] Murai K. (2017). “The application to maritime society of patch-type device,” in 2017 6th International Conference on Informatics, Electronics and Vision andamp; 2017 7th International Symposium in Computational Medical and Health Technology (ICIEV-ISCMHT). Available online at: https://doi.ieeecomputersociety.org/10.1109/ICIEV.2017.8338516 (accessed March 14, 2023).

[B94] Murai K. Hayashi Y. Okazaki T. Stone L. C. Nobuo M. (2008). “Evaluation of ship navigator's mental workload using nasal temperature and heart rate variability,” in 2008 IEEE International Conference on Systems, Man and Cybernetics (Singapore: IEEE). 10.1109/ICSMC.2008.4811503

[B95] Murai K. Wang J. Wang Y. Qilei Y. (2017). “Toward evaluation of mixed culture's team works: case study of ship bridge simulator-based training for cadets,” in 2017 Joint 17th World Congress of International Fuzzy Systems Association and 9th International Conference on Soft Computing and Intelligent Systems (IFSA-SCIS) (Himeji: IEEE). 10.1109/IFSA-SCIS.2017.8023343

[B96] Murata A. Iwase H. (2000). Evaluation of mental workload by variability of pupil area. IEICE Trans. Inform. Syst. E83-D, 1187–1190.

[B97] Murray I. R. Baber C. South A. (1996). Towards a definition and working model of stress and its effects on speech. Speech Commun. 20, 3–12. 10.1016/S0167-6393(96)00040-427534393

[B98] Naik R. Kogkas A. ros Ashrafian H. Mylonas G. Darzi A. (2022). The measurement of cognitive workload in surgery using pupil metrics: a systematic review and narrative analysis. J. Surg. Res. 280, 258–272. 10.1016/j.jss.2022.07.010 36030601

[B99] Naismith L. M. Cavalcanti R. B. (2015). Validity of cognitive load measures in simulation-based training: a systematic review. Acad. Med. 90, S24–35. 10.1097/ACM.0000000000000893 26505098

[B100] Nater U. M. Rohleder N. (2009). Salivary alpha-amylase as a non-invasive biomarker for the sympathetic nervous system: current state of research. Psychoneuroendocrinology 34, 486–496. 10.1016/j.psyneuen.2009.01.014 19249160

[B101] O'Donnell R. D. Eggemeier F. T. (1986). “Workload assessment methodology,” in Handbook of Perception and Human Performance, Vol. 2. Cognitive Processes and Performance, eds. Boff K. R. Kaufman L. Thomas J. P. (Oxford: John Wiley & Sons), 1–49.

[B102] Or C. K. L. Duffy V. G. (2007). Development of a facial skin temperature-based methodology for non-intrusive mental workload measurement. Occup. Ergon. 7, 83–94. 10.3233/OER-2007-7202

[B103] Ouzzani M. Hammady H. Fedorowicz Z. Elmagarmid A. (2016). Rayyan—a web and mobile app for systematic reviews. Syst. Rev. 5:210. 10.1186/s13643-016-0384-4 27919275 PMC5139140

[B104] Page M. J. McKenzie J. E. Bossuyt P. M. Boutron I. Hoffmann T. C. Mulrow C. D. . (2021). The PRISMA 2020 statement: an updated guideline for reporting systematic reviews. BMJ 372:n71. 10.1136/bmj.n71 33782057 PMC8005924

[B105] Parasuraman R. Sheridan T. B. Wickens C. D. (2008). Situation awareness, mental workload, and trust in automation: viable, empirically supported cognitive engineering constructs. J. Cogn. Eng. Decision Making 2, 140–160. 10.1518/155534308X284417

[B106] Patil V. P. Nayak K. K. Saxena M. (2013). Voice stress detection. Int. J. Electr. Electron. Comput. Eng. 2, 148–154.

[B107] Paxion J. Galy E. Berthelon C. (2014). Mental workload and driving. Front. Psychol. 5:1344. 10.3389/fpsyg.2014.01344 25520678 PMC4251303

[B108] Peruzzini M. Grandi F. Pellicciari M. (2017). “Benchmarking of tools for user eXperience analysis in Industry 4.0,” in 27th International Conference on Flexible Automation and Intelligent Manufacturing, Faim2017 (Modena: Elsevier), Vol. 11, 806–813. 10.1016/j.promfg.2017.07.182

[B109] Peruzzini M. Grandi F. Pellicciari M. (2020). Exploring the potential of Operator 4.0 interface and monitoring. Comput. Indus. Eng. 139:105600. 10.1016/j.cie.2018.12.04739160841

[B110] Podsakoff P. M. MacKenzie S. B. Lee J. Y. Podsakoff N. P. (2003). Common method biases in behavioral research: a critical review of the literature and recommended remedies. J. Appl. Psychol. 88, 879–903. 10.1037/0021-9010.88.5.879 14516251

[B111] Pozzi D. Matteoli M. (2018). The hypothalamic-LC-PFC axis: a new “ace” in the brain for fast-behavioral stress response. EMBO J. 37:e100702. 10.15252/embj.2018100702 30361465 PMC6213267

[B112] Qu X. (2013). Effects of cognitive and physical loads on local dynamic stability during gait. Appl. Ergon. 44, 455–458. 10.1016/j.apergo.2012.10.018 23176787

[B113] Rodríguez S. Sánchez L. López P. Cañas J. J. (2015). “Pupillometry to assess air traffic controller workload through the mental workload model,” in Proceedings of the 5th International Conference on Application and Theory of Automation in Command and Control Systems, Toulouse, France (New York, NY: Association for Computing Machinery). 10.1145/2899361.2899371

[B114] Roscoe A. H. (1992). Assessing pilot workload. Why measure heart rate, HRV and respiration? Biol. Psychol. 34, 259–287. 10.1016/0301-0511(92)90018-P 1467396

[B115] Rusnock C. F. Borghetti B. J. (2018). Workload profiles: a continuous measure of mental workload. Int. J. Indus. Ergon. 63, 49–64. 10.1016/j.ergon.2016.09.003

[B116] Saedi S. Fini A. A. F. Khanzadi M. Wong J. Sheikhkhoshkar M. Banaei M. (2022). Applications of electroencephalography in construction. Automat. Construct. 133:103985. 10.1016/j.autcon.2021.103985

[B117] Sanders A. F. (1983). Towards a model of stress and human performance. Acta Psychol. 53, 61–97. 10.1016/0001-6918(83)90016-1 6869047

[B118] Scherer K. Grandjean D. Johnstone T. Klasmeyer G. Bänziger T. (2002). “Acoustic correlates of task load and stress,” in Proceedings of 7th International Conference on Spoken Language Processing (ICSLP 2002) (Denver, CO: International Speech Communication Association), 2017–2020. 10.21437/ICSLP.2002-554

[B119] Setz C. Arnrich B. Schumm J. La Marca R. Tröster G. Ehlert U. (2010). Discriminating stress from cognitive load using a wearable EDA device. IEEE Trans. Inform. Technonol. Biomed. 14, 410–417. 10.1109/TITB.2009.2036164 19906598

[B120] Shah P. Khaleel M. Thuptimdang W. Sunwoo J. Veluswamy S. Chalacheva P. . (2020). Mental stress causes vasoconstriction in subjects with sickle cell disease and in normal controls. Haematologica 105, 83–90. 10.3324/haematol.2018.211391 30975906 PMC6939522

[B121] Shakouri M. Ikuma L. H. Aghazadeh F. Nahmens I. (2018). Analysis of the sensitivity of heart rate variability and subjective workload measures in a driving simulator: the case of highway work zones. Int. J. Indus. Ergon. 66, 136–145. 10.1016/j.ergon.2018.02.015

[B122] Shayesteh S. Ojha A. Liu Y. Jebelli H. (2023). Human-robot teaming in construction: evaluative safety training through the integration of immersive technologies and wearable physiological sensing. Saf. Sci. 159:106019. 10.1016/j.ssci.2022.106019

[B123] Solhjoo S. Haigney M. C. McBee E. van Merrienboer J. J. G. Schuwirth L. Artino A. R. . (2019). Heart rate and heart rate variability correlate with clinical reasoning performance and self-reported measures of cognitive load. Sci. Rep. 9:14668. 10.1038/s41598-019-50280-3 31604964 PMC6789096

[B124] Solovey E. T. Girouard A. Chauncey K. Hirshfield L. M. Sassaroli A. Zheng F. . (2009). “Using fNIRS brain sensing in realistic HCI settings: experiments and guidelines,” in Proceedings of the 22nd Annual ACM Symposium on User Interface Software and Technology, Victoria, BC, Canada (New York, NY: Association for Computing Machinery). 10.1145/1622176.1622207

[B125] Srinivasan R. Srinivasan B. Iqbal M. U. Nemet A. Kravanja Z. (2019). Recent developments towards enhancing process safety: inherent safety and cognitive engineering. Comput. Chem. Eng. 128, 364–383. 10.1016/j.compchemeng.2019.05.034

[B126] Sriranga A. K. Lu Q. Birrell S. (2023). A systematic review of in-vehicle physiological indices and sensor technology for driver mental workload monitoring. Sensors 23:2214. 10.3390/s23042214 36850812 PMC9963326

[B127] Stanton N. A. Hedge A. Brookhuis K. Salas E. Hendrick H. W. (2004). Handbook of Human Factors and Ergonomics Methods. Boca Raton, FL: CRC Press. 10.1201/9780203489925

[B128] Strangman G. Boas D. A. Sutton J. P. (2002). Non-invasive neuroimaging using near-infrared light. Biol. Psychiatry 52, 679–693. 10.1016/S0006-3223(02)01550-0 12372658

[B129] Tao D. Tan H. Wang H. Zhang X. Qu X. Zhang T. (2019). A systematic review of physiological measures of mental workload. Int. J. Environ. Res. Public Health 16:2716. 10.3390/ijerph16152716 31366058 PMC6696017

[B130] Tattersall A. J. Foord P. S. (1996). An experimental evaluation of instantaneous self-assessment as a measure of workload. Ergonomics 39, 740–748. 10.1080/00140139608964495 8635447

[B131] Tsai Y. F. Viirre E. Strychacz C. Chase B. Jung T. P. (2007). Task performance and eye activity: predicting behavior relating to cognitive workload. Aviat. Space Environ. Med. 78, B176–185. 17547318

[B132] Tuscan L.-A. Herbert J. D. Forman E. M. Juarascio A. S. Izzetoglu M. Schultheis M. (2013). Exploring frontal asymmetry using functional near-infrared spectroscopy: a preliminary study of the effects of social anxiety during interaction and performance tasks. Brain Imag. Behav. 7, 140–153. 10.1007/s11682-012-9206-z 23132684

[B133] Ulutas B. H. Firat Ozkan N. (2019). Assessing occupational risk factors for forklift drivers. Le travail humain 82, 129–149. 10.3917/th.822.012918052372

[B134] Umer W. (2022). Simultaneous monitoring of physical and mental stress for construction tasks using physiological measures. J. Build. Eng. 46:103777. 10.1016/j.jobe.2021.103777

[B135] Veltman J. A. Gaillard A. W. K. (1996). Physiological indices of workload in a simulated flight task. Biol. Psychol. 42, 323–342. 10.1016/0301-0511(95)05165-1 8652751

[B136] Wallin B. G. (1981). Sympathetic nerve activity underlying electrodermal and cardiovascular reactions in man. Psychophysiology 18, 470–476. 10.1111/j.1469-8986.1981.tb02483.x 7267931

[B137] Wang L. Gao S. Tan W. Zhang J. (2022). Pilots' mental workload variation when taking a risk in a flight scenario: a study based on flight simulator experiments. Int. J. Occup. Saf. Ergon. 29, 366–375. 10.1080/10803548.2022.2049101 35236244

[B138] Westbrook A. Braver T. S. (2016). Dopamine does double duty in motivating cognitive effort. Neuron 89, 695–710. 10.1016/j.neuron.2015.12.029 26889810 PMC4759499

[B139] Whitmore J. Fisher S. (1996). Speech during sustained operations. Speech Commun. 20, 55–70. 10.1016/S0167-6393(96)00044-1

[B140] Wickens C. D. (2008). Multiple resources and mental workload. Hum. Fact. 50, 449–455. 10.1518/001872008X288394 18689052

[B141] Wijsman J. Grundlehner B. Penders J. Hermens H. (2010). “Trapezius muscle EMG as predictor of mental stress,” in Wireless Health 2010, San Diego, California. 10.1145/1921081.1921100

[B142] Wilbanks B. A. McMullan S. P. (2018). A review of measuring the cognitive workload of electronic health records. Comput. Inform. Nurs. 36, 579–588. 10.1097/CIN.0000000000000469 30134256

[B143] Wilson G. F. (1992). Applied use of cardiac and respiration measures: practical considerations and precautions. Biol. Psychol. 34, 163–178. 10.1016/0301-0511(92)90014-L 1467392

[B144] Wittels P. Johannes B. Enne R. Kirsch K. Gunga H. C. (2002). Voice monitoring to measure emotional load during short-term stress. Euro. J. Appl. Physiol. 87, 278–282. 10.1007/s00421-002-0625-1 12111290

[B145] Wittenberg C. (2015). “Cause the trend Industry 4.0 in the automated industry to new requirements on user interfaces?,” in Human-Computer Interaction: Users and Contexts, ed. Kurosu M. (Cham: Springer), 238–245. 10.1007/978-3-319-21006-3_24

[B146] Womack B. D. Hansen J. H. L. (1999). N-channel hidden Markov models for combined stressed speech classification and recognition. IEEE Trans. Speech Audio Process. 7, 668–677. 10.1109/89.799692

[B147] Wu C. Cha J. Sulek J. Sundaram C. P r. . (2021). Sensor-based indicators of performance changes between sessions during robotic surgery training. Appl. Ergon. 90:103251. 10.1016/j.apergo.2020.103251 32961465 PMC7606790

[B148] Xing X. Ma Z. Zhang M. Zhou Y. Dong W. Song M. (2019). An unobtrusive and calibration-free blood pressure estimation method using photoplethysmography and biometrics. Sci. Rep. 9:8611. 10.1038/s41598-019-45175-2 31197243 PMC6565722

[B149] Yerkes R. M. Dodson J. D. (1908). The relation of strength of stimulus to rapidity of habit-formation. J. Compar. Neurol. Psychol. 18, 459–482. 10.1002/cne.920180503

[B150] Ying L. Fu S. Qian X. Sun X. (2011). Effects of mental workload on long-latency auditory-evoked-potential, salivary cortisol, and immunoglobulin A. Neurosci. Lett. 491, 31–34. 10.1016/j.neulet.2011.01.002 21215297

[B151] Young M. S. Brookhuis K. A. Wickens C. D. Hancock P. A. (2015). State of science: mental workload in ergonomics. Ergonomics 58, 1–17. 10.1080/00140139.2014.956151 25442818

[B152] Zhang X. Mahadevan S. Lau N. Weinger M. B. (2020). Multi-source information fusion to assess control room operator performance. Reliabil. Eng. Syst. Saf. 194:106287. 10.1016/j.ress.2018.10.012

[B153] Zheng B. Jiang X. Tien G. Meneghetti A. Panton O. N. Atkins M. S. (2012). Workload assessment of surgeons: correlation between NASA TLX and blinks. Surg. Endosc. 26, 2746–2750. 10.1007/s00464-012-2268-6 22527300

[B154] Zheng T. Glock C. H. Grosse E. H. (2022). Opportunities for using eye tracking technology in manufacturing and logistics: systematic literature review and research agenda. Comput. Indus. Eng. 171:108444. 10.1016/j.cie.2022.108444

[B155] Zoaktafi M. Kazemi R. Choobineh A. SaboorYaraghi A. Nematolahi S. Zakerian S. A. (2020). Relationship between mental workload and salivary cortisol levels: a field study. Work 67, 381–386. 10.3233/WOR-203287 33044218

